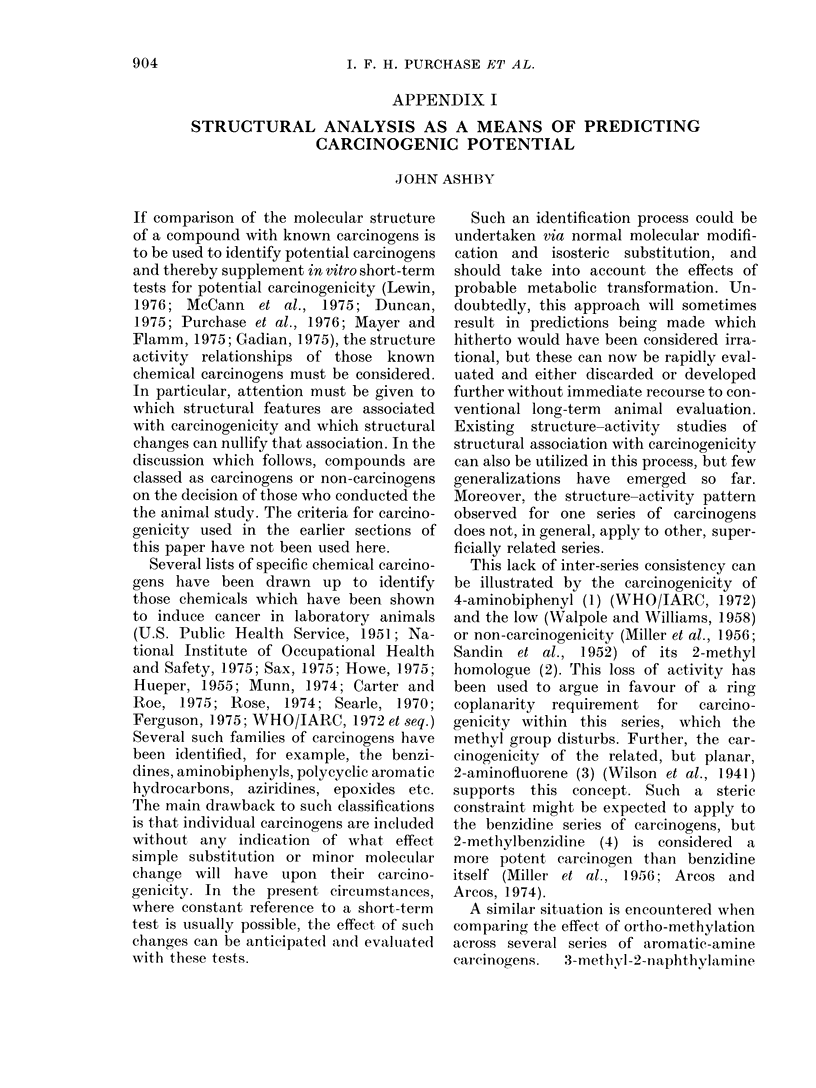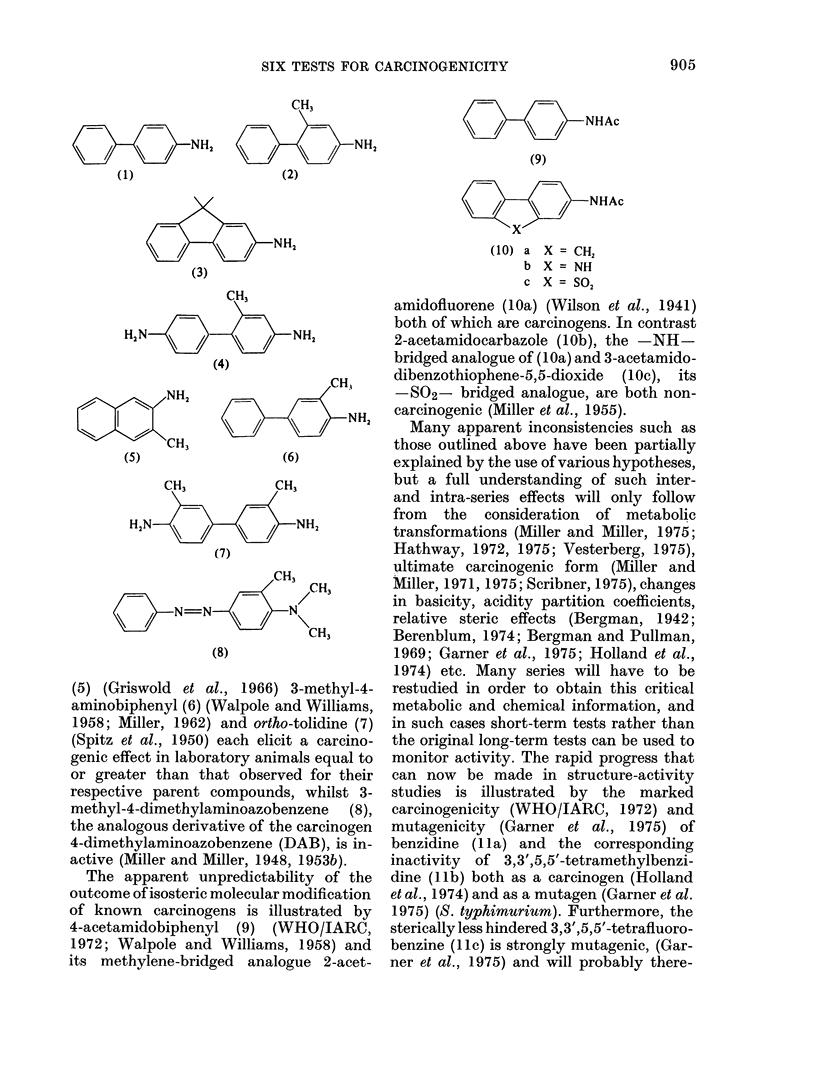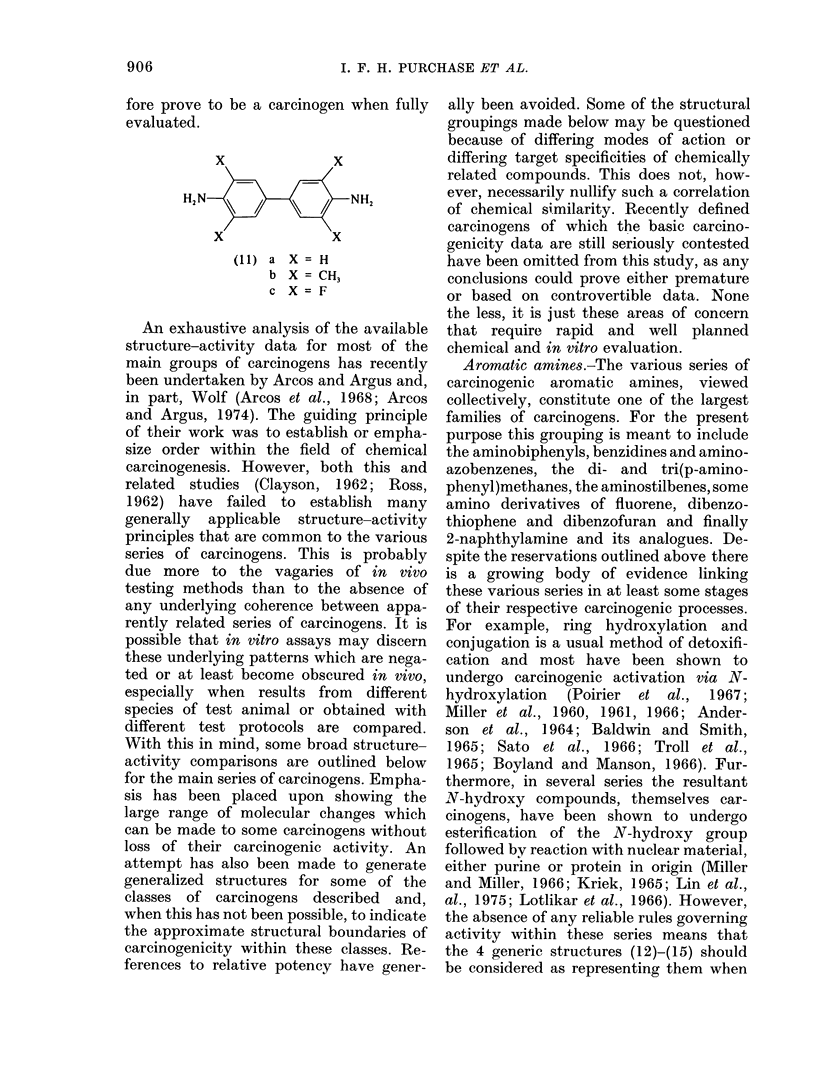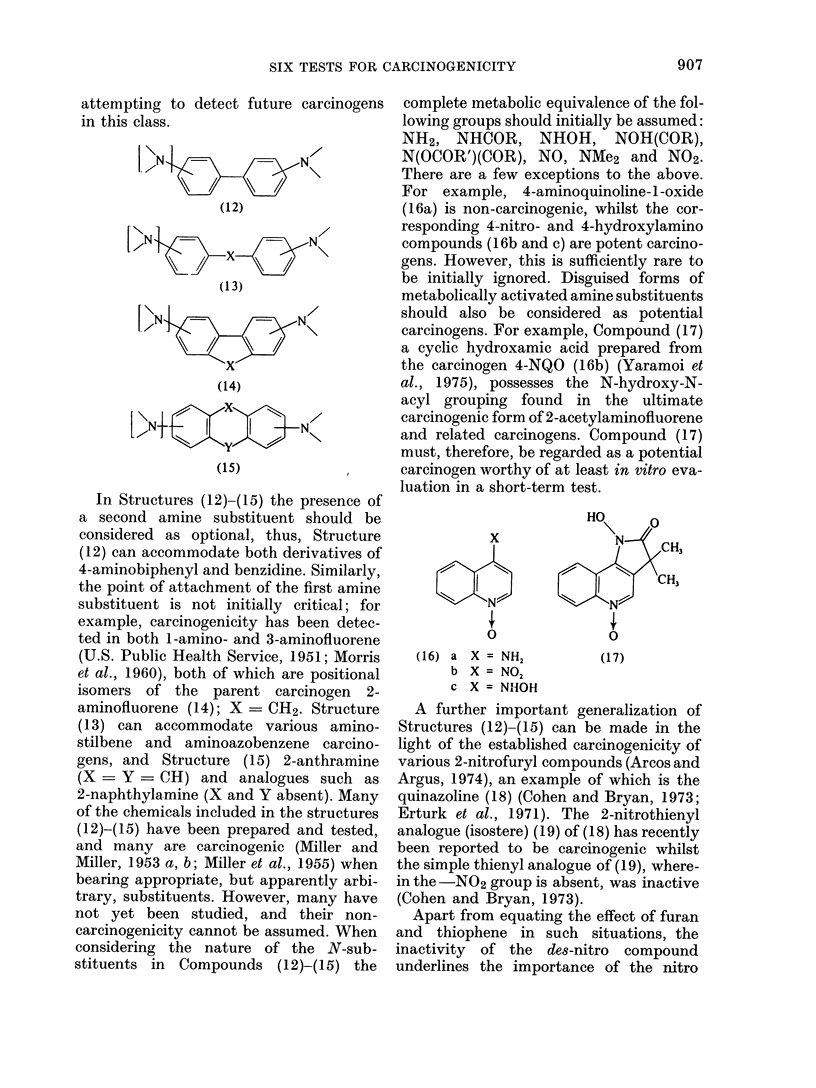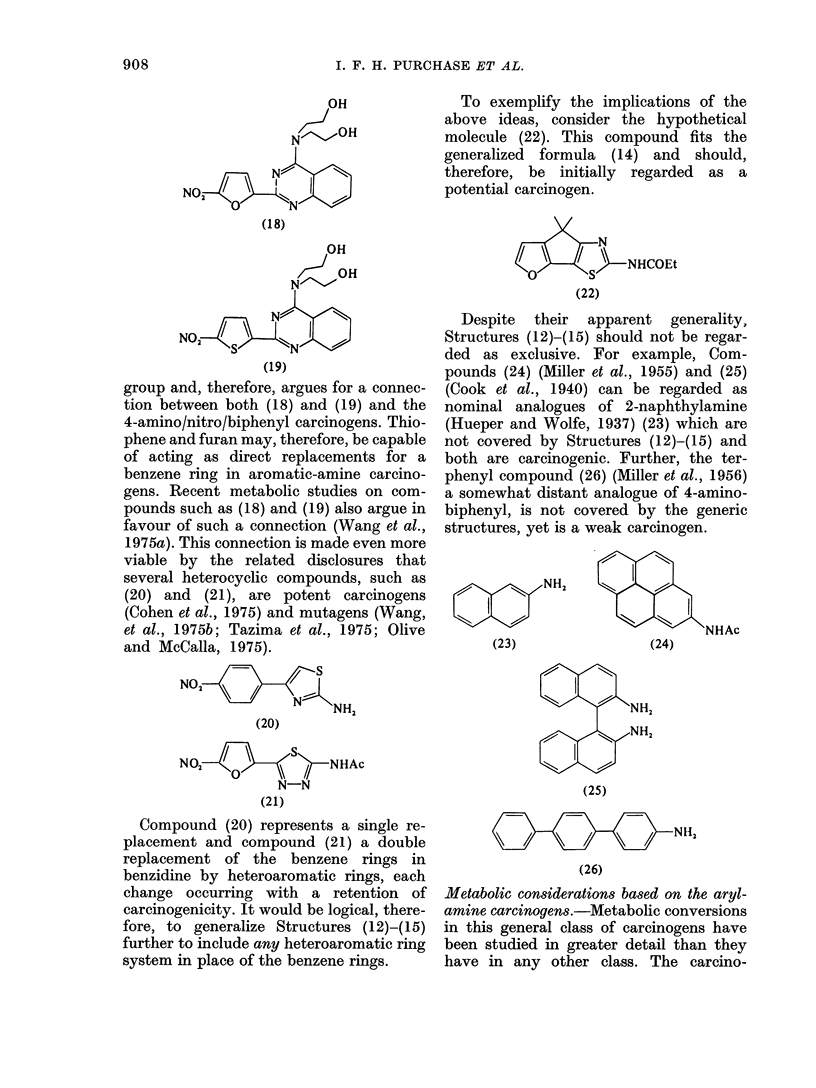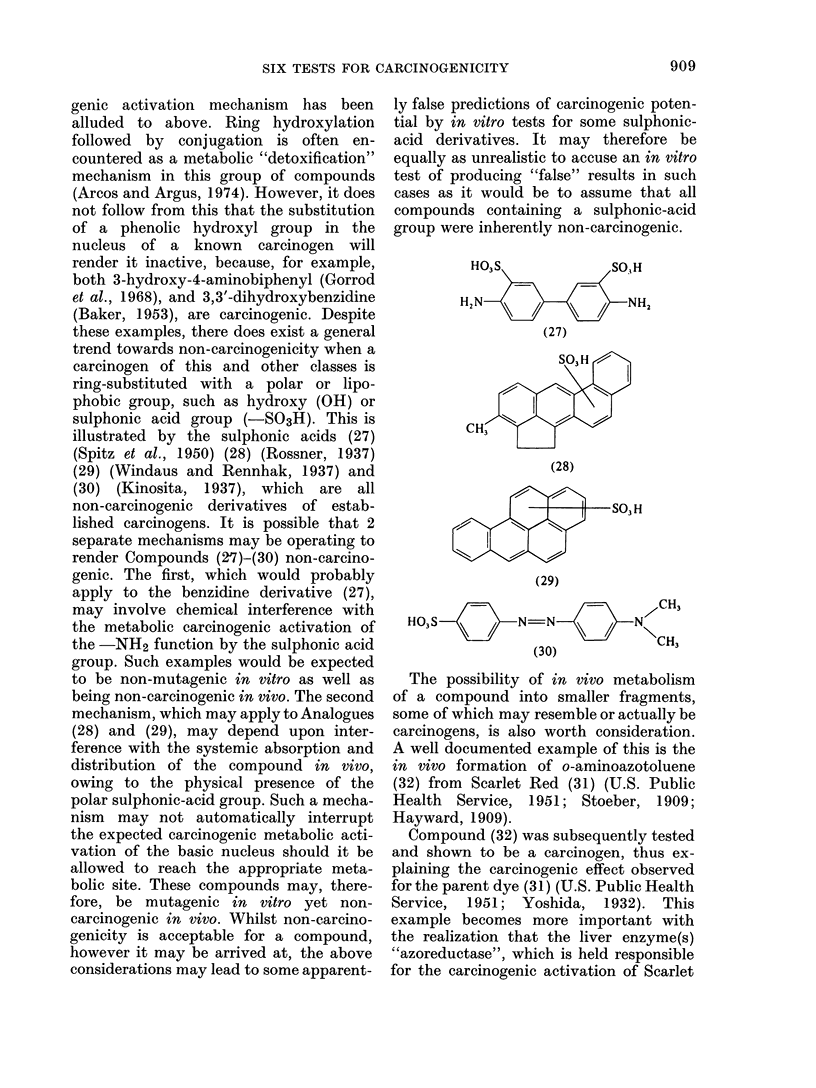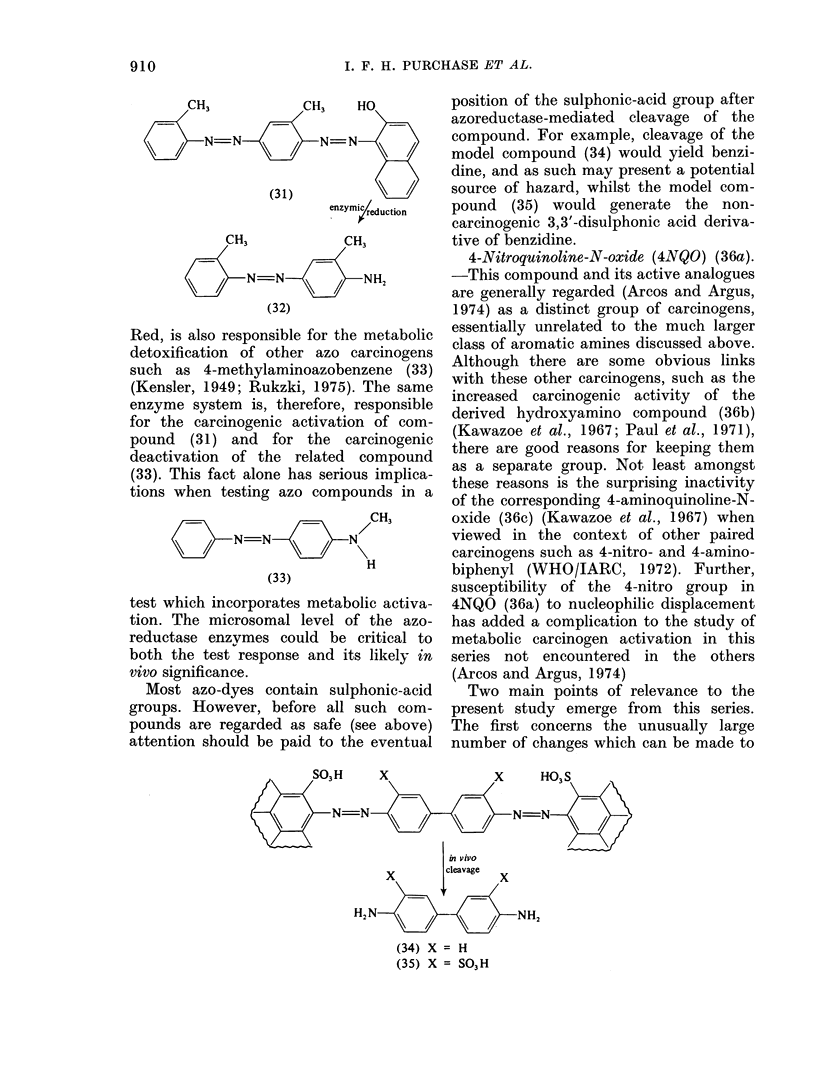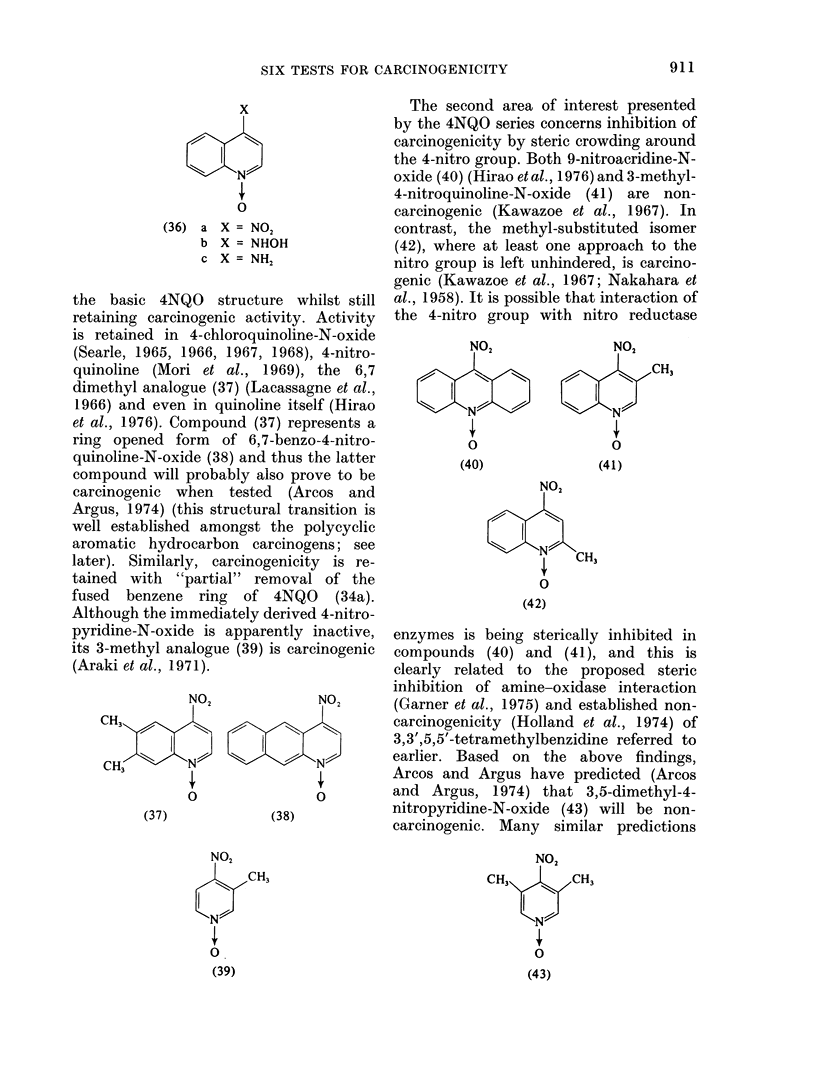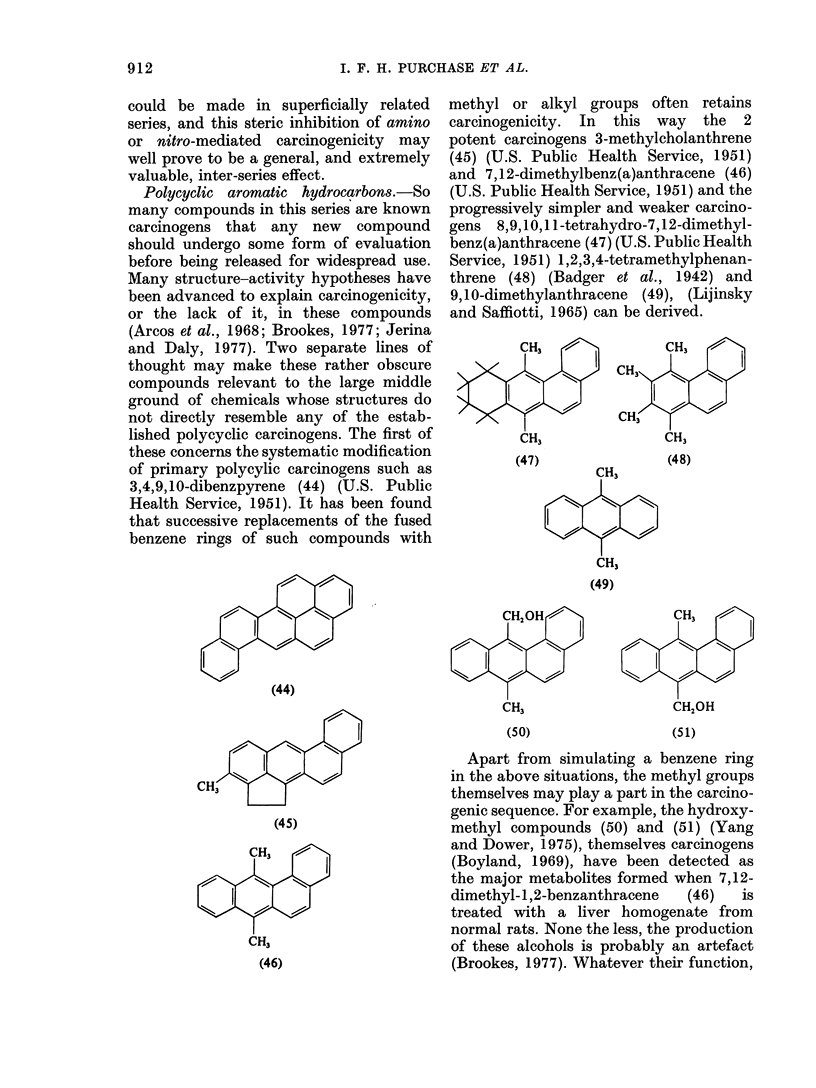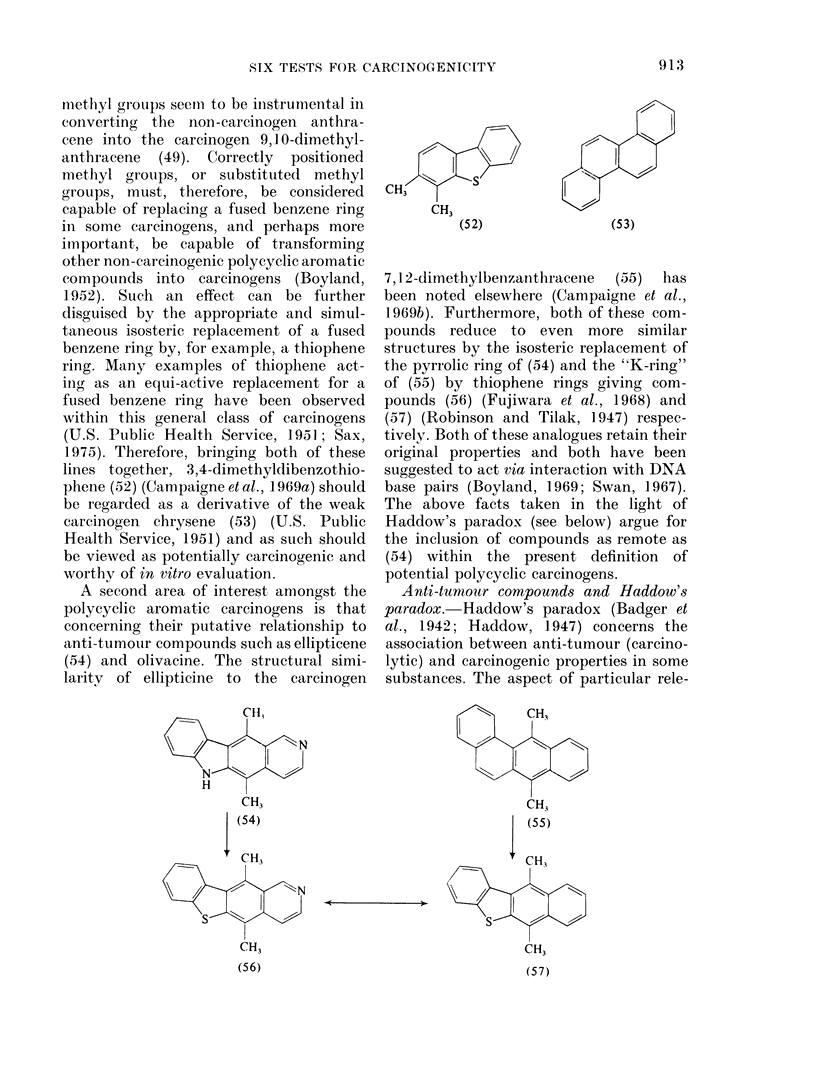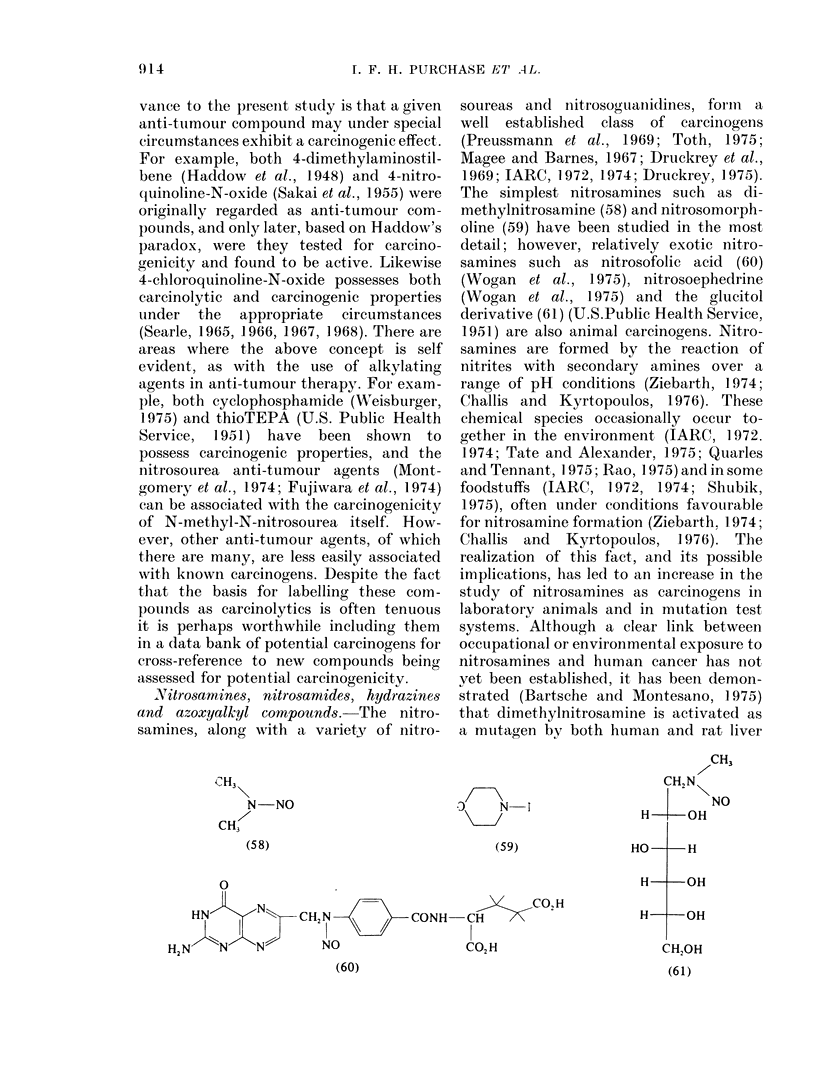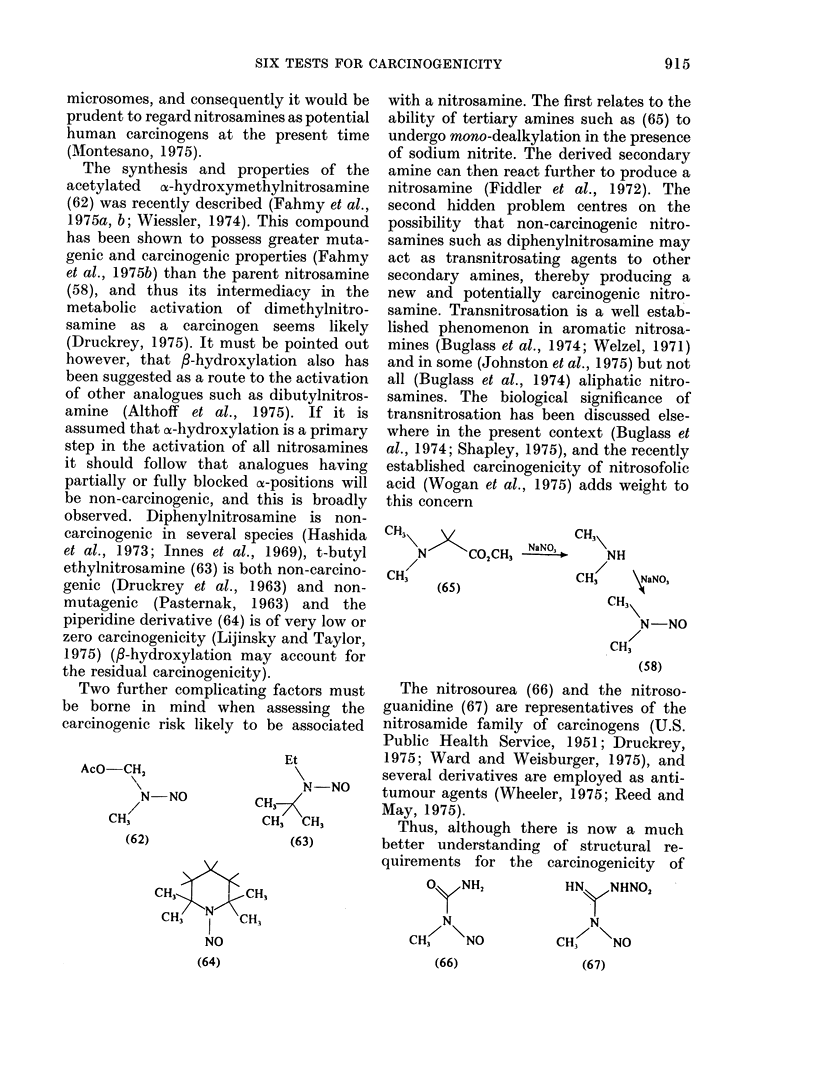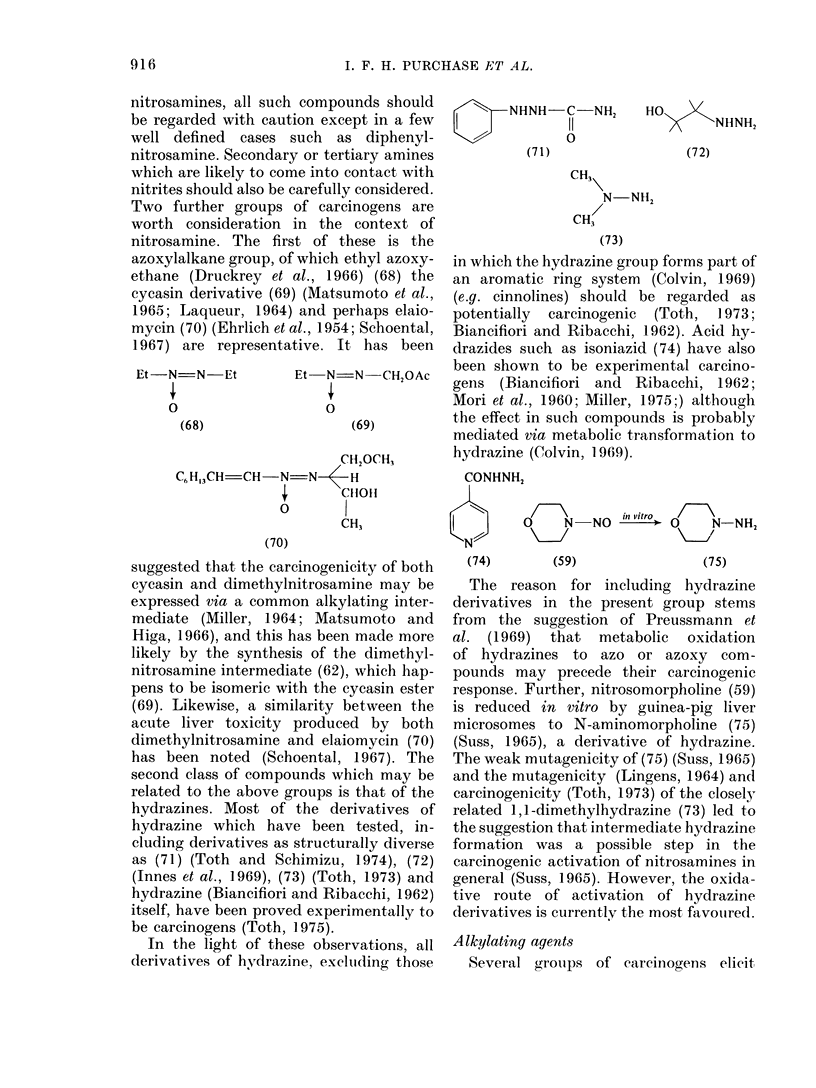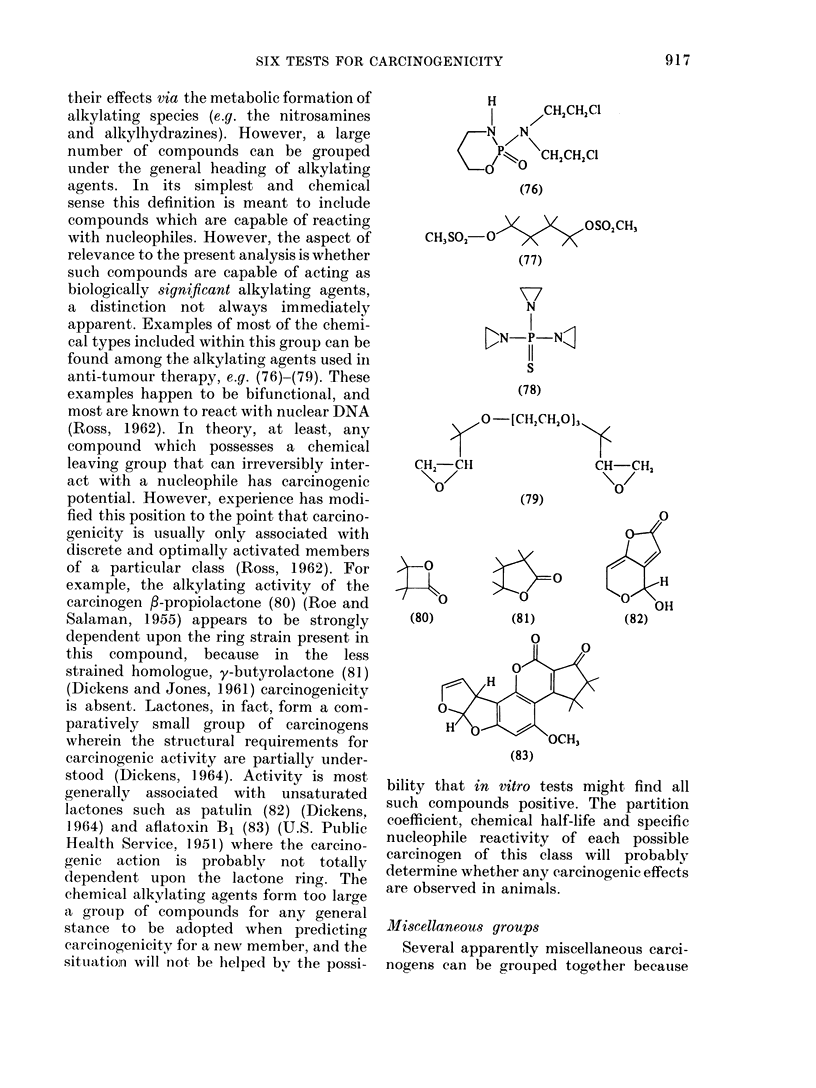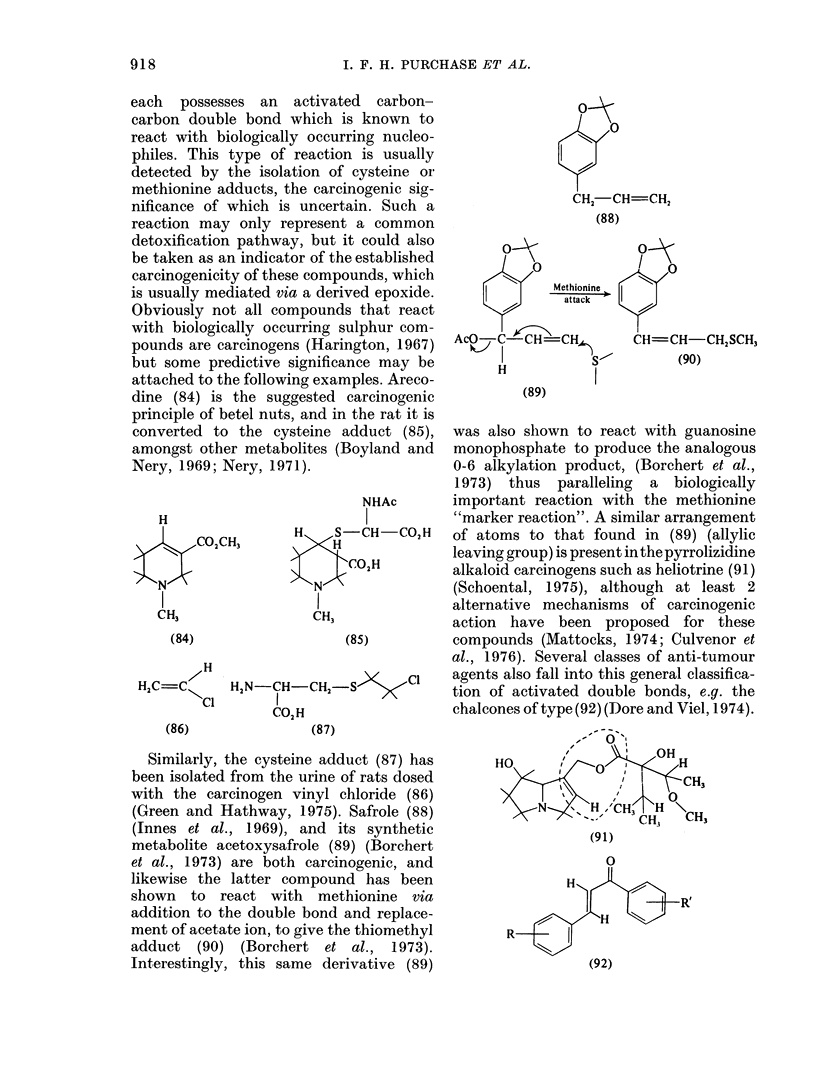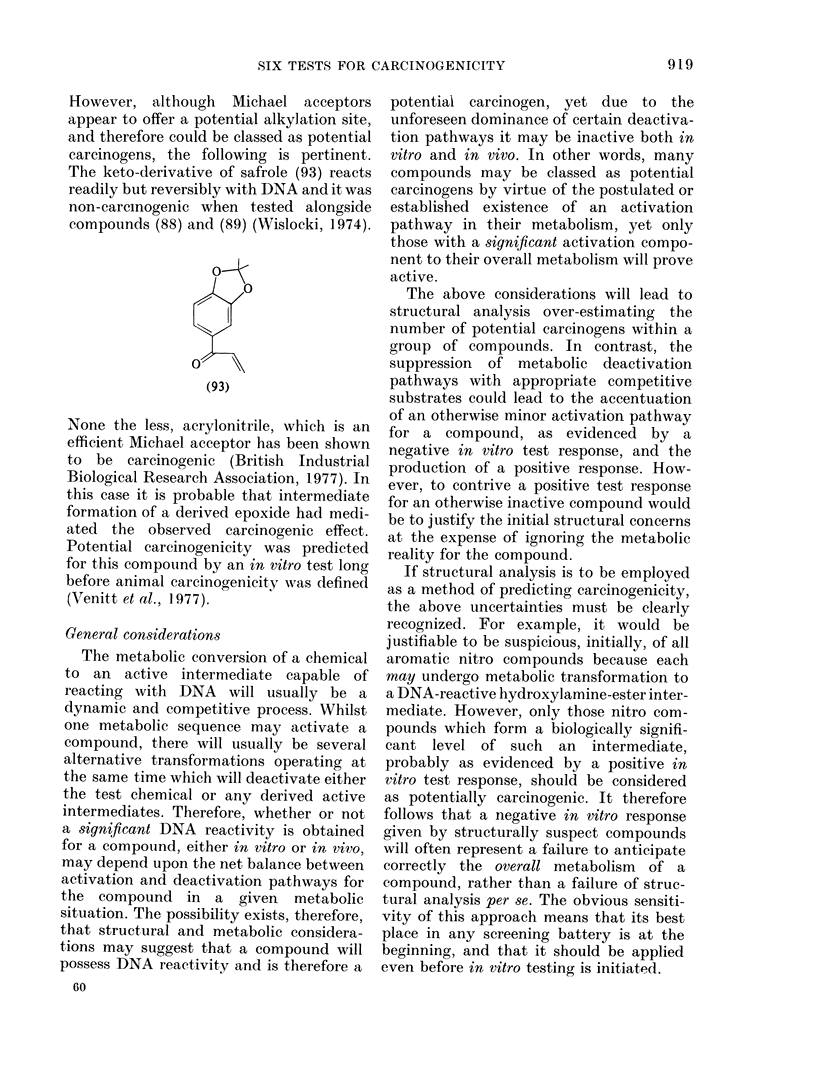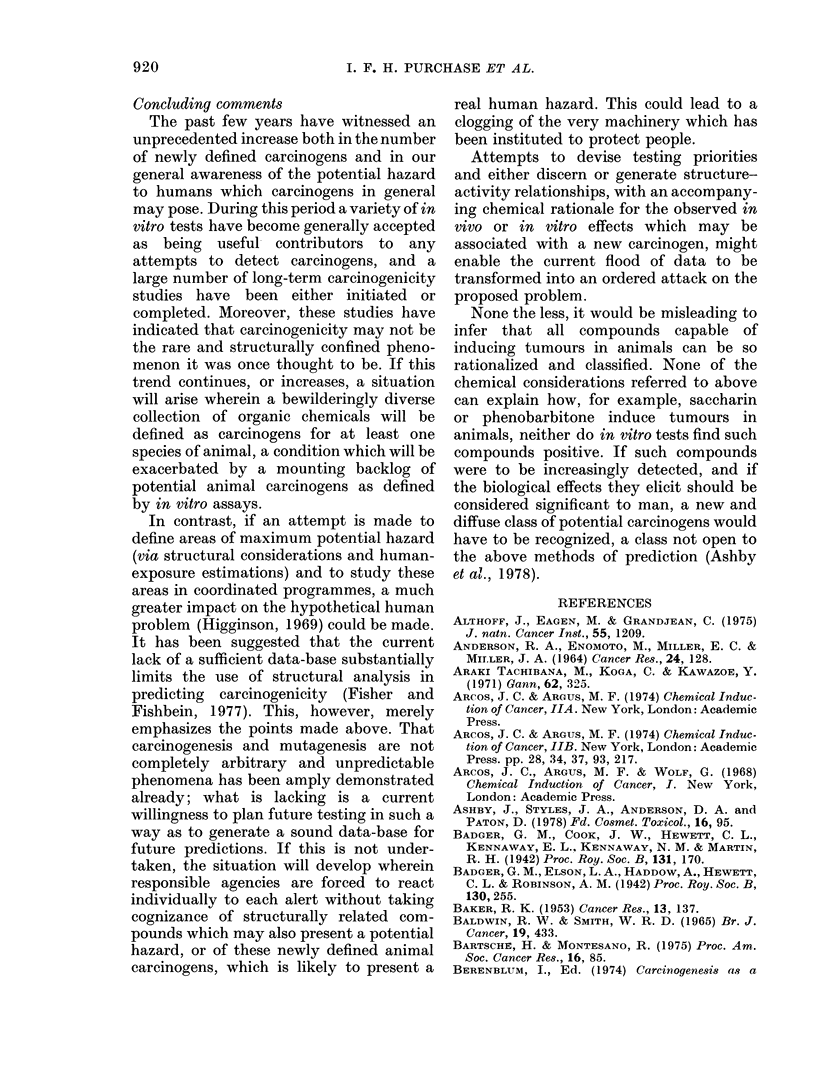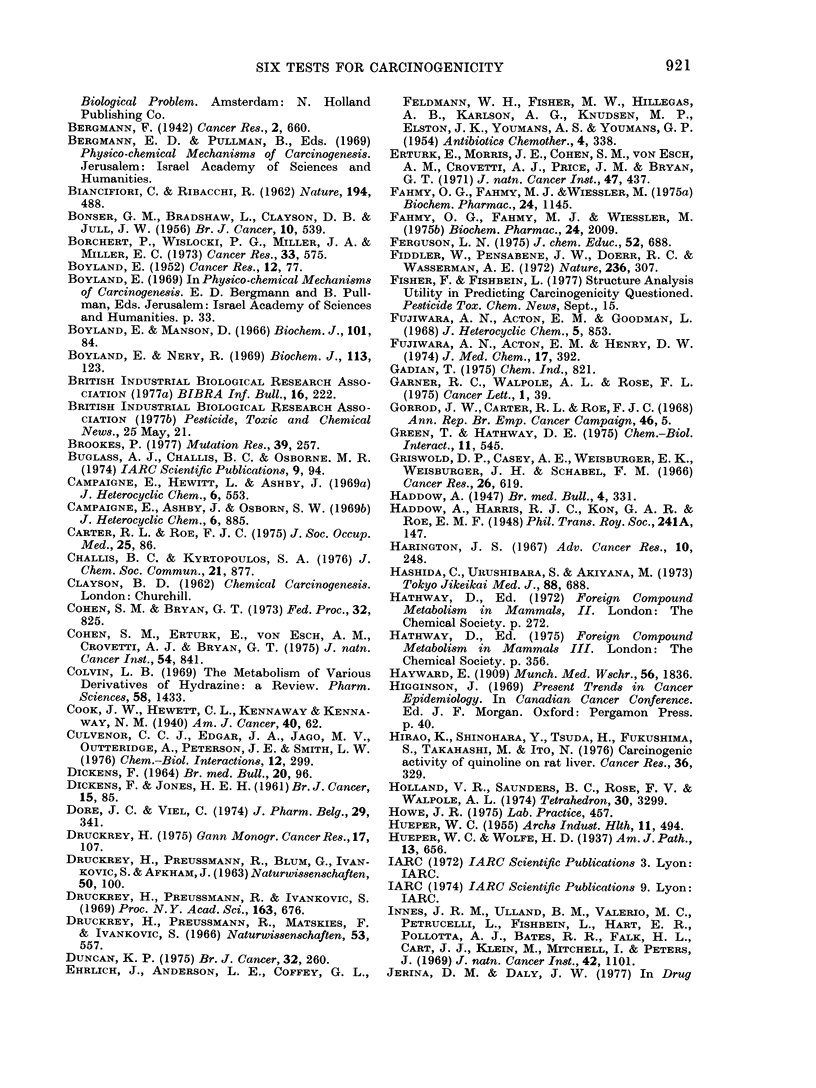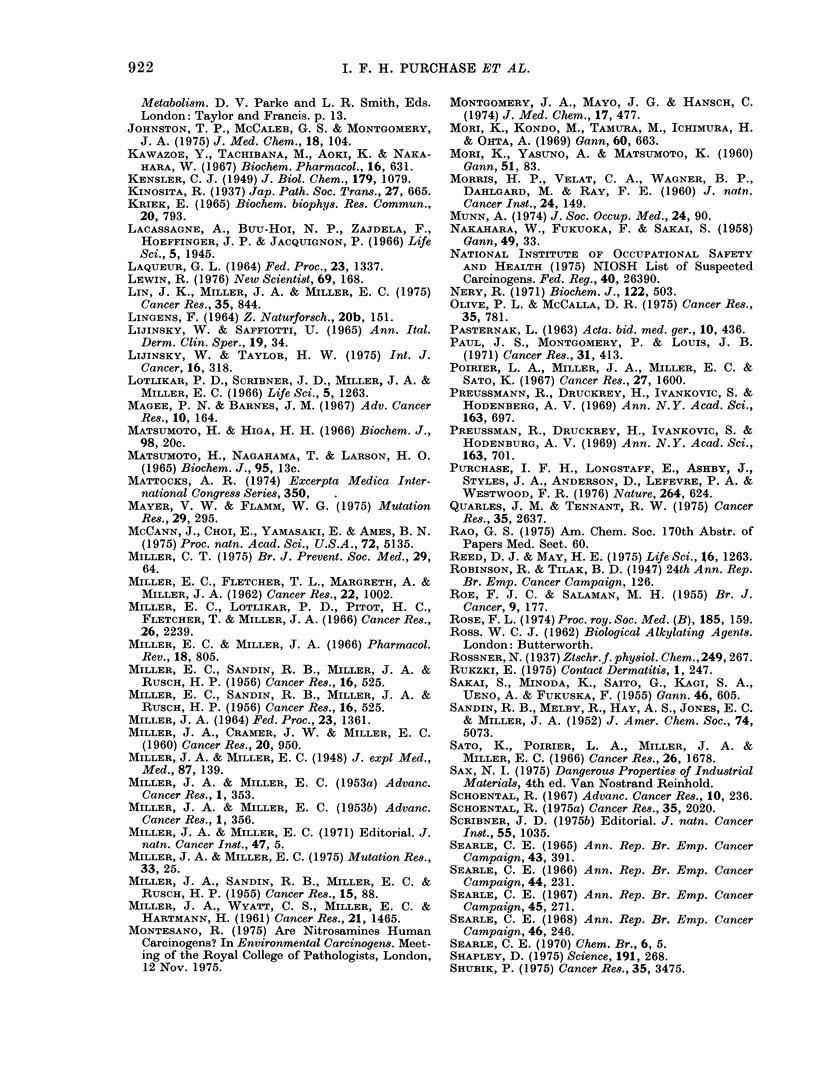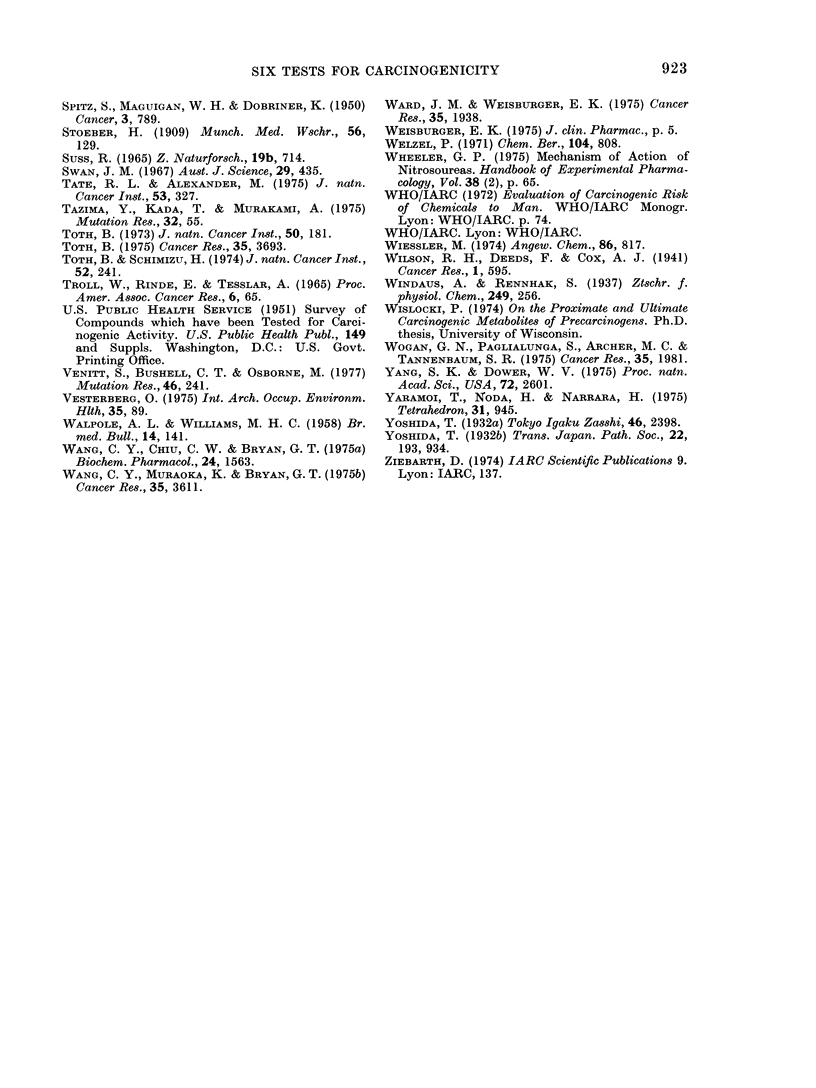# Structural analysis as a means of predicting carcinogenic potential.

**DOI:** 10.1038/bjc.1978.133

**Published:** 1978-06

**Authors:** J. Ashby


### APPENDIX I

### STRUCTURAL ANALYSIS AS A MEANS OF PREDICTING CARCINOGENIC POTENTIAL

JOHN ASHBY

If comparison of the molecular structure of a compound with known carcinogens is to be used to identify potential carcinogens and thereby supplement *in vitro* short-term tests for potential carcinogenicity (Lewin, 1976; McCann *et al.*, 1975; Duncan, 1975; Purchase *et al.*, 1976; Mayer and Flamm, 1975; Gadian, 1975), the structure activity relationships of those known chemical carcinogens must be considered. In particular, attention must be given to which structural features are associated with carcinogenicity and which structural changes can nullify that association. In the discussion which follows, compounds are classed as carcinogens or non-carcinogens on the decision of those who conducted the the animal study. The criteria for carcinogenicity used in the earlier sections of this paper have not been used here.

Several lists of specific chemical carcinogens have been drawn up to identify those chemicals which have been shown to induce cancer in laboratory animals (U.S. Public Health Service, 1951; National Institute of Occupational Health and Safety, 1975; Sax, 1975; Howe, 1975; Hueper, 1955; Munn, 1974; Carter and Roe, 1975; Rose, 1974; Searle, 1970; Ferguson, 1975; WHO/IARC, 1972 *et seq.*) Several such families of carcinogens have been identified, for example, the benzidines, aminobiphenyls, polycyclic aromatic hydrocarbons, aziridines, epoxides etc. The main drawback to such classifications is that individual carcinogens are included without any indication of what effect simple substitution or minor molecular change will have upon their carcinogenicity. In the present circumstances, where constant reference to a short-term test is usually possible, the effect of such changes can be anticipated and evaluated with these tests.

Such an identification process could be undertaken *via* normal molecular modification and isosteric substitution, and should take into account the effects of probable metabolic transformation. Undoubtedly, this approach will sometimes result in predictions being made which hitherto would have been considered irrational, but these can now be rapidly evaluated and either discarded or developed further without immediate recourse to conventional long-term animal evaluation. Existing structure–activity studies of structural association with carcinogenicity can also be utilized in this process, but few generalizations have emerged so far. Moreover, the structure–activity pattern observed for one series of carcinogens does not, in general, apply to other, superficially related series.

This lack of inter-series consistency can be illustrated by the carcinogenicity of 4-aminobiphenyl (1) (WHO/IARC, 1972) and the low (Walpole and Williams, 1958) or non-carcinogenicity (Miller *et al.*, 1956; Sandin *et al.*, 1952) of its 2-methyl homologue (2). This loss of activity has been used to argue in favour of a ring coplanarity requirement for carcinogenicity within this series, which the methyl group disturbs. Further, the carcinogenicity of the related, but planar, 2-aminofluorene (3) (Wilson *et al.*, 1941) supports this concept. Such a steric constraint might be expected to apply to the benzidine series of carcinogens, but 2-methylbenzidine (4) is considered a more potent carcinogen than benzidine itself (Miller *et al.*, 1956; Arcos and Arcos, 1974).

A similar situation is encountered when comparing the effect of ortho-methylation across several series of aromatic-amine carcinogens. 3-methyl-2-naphthylamine

(1)

(2)

(3)

(4)

(5)

(6)

(7)

(8)

(5) (Griswold *et al.*, 1966) 3-methyl-4-aminobiphenyl (6) (Walpole and Williams, 1958; Miller, 1962) and *ortho*-tolidine (7) (Spitz *et al.*, 1950) each elicit a carcinogenic effect in laboratory animals equal to or greater than that observed for their respective parent compounds, whilst 3-methyl-4-dimethylaminoazobenzene (8), the analogous derivative of the carcinogen 4-dimethylaminoazobenzene (DAB), is inactive (Miller and Miller, 1948, 1953*b*).

The apparent unpredictability of the outcome of isosteric molecular modification of known carcinogens is illustrated by 4-acetamidobiphenyl (9) (WHO/IARC, 1972; Walpole and Williams, 1958) and its methylene-bridged analogue 2-acet-

(9)

(10) a X = $CH_2$
b X = NH
c X = $SO_2$

amidofluorene (10a) (Wilson *et al.*, 1941) both of which are carcinogens. In contrast 2-acetamidocarbazole (10b), the —NH— bridged analogue of (10a) and 3-acetamidodibenzothiophene-5,5-dioxide (10c), its —$SO_2$— bridged analogue, are both non-carcinogenic (Miller *et al.*, 1955).

Many apparent inconsistencies such as those outlined above have been partially explained by the use of various hypotheses, but a full understanding of such inter- and intra-series effects will only follow from the consideration of metabolic transformations (Miller and Miller, 1975; Hathway, 1972, 1975; Vesterberg, 1975), ultimate carcinogenic form (Miller and Miller, 1971, 1975; Scribner, 1975), changes in basicity, acidity partition coefficients, relative steric effects (Bergman, 1942; Berenblum, 1974; Bergman and Pullman, 1969; Garner *et al.*, 1975; Holland *et al.*, 1974) etc. Many series will have to be restudied in order to obtain this critical metabolic and chemical information, and in such cases short-term tests rather than the original long-term tests can be used to monitor activity. The rapid progress that can now be made in structure-activity studies is illustrated by the marked carcinogenicity (WHO/IARC, 1972) and mutagenicity (Garner *et al.*, 1975) of benzidine (11a) and the corresponding inactivity of 3,3′,5,5′-tetramethylbenzidine (11b) both as a carcinogen (Holland *et al.*, 1974) and as a mutagen (Garner *et al.* 1975) (*S. typhimurium*). Furthermore, the sterically less hindered 3,3′,5,5′-tetrafluorobenzine (11c) is strongly mutagenic, (Garner *et al.*, 1975) and will probably therefore prove to be a carcinogen when fully evaluated.

(11) a X = H
b X = $CH_3$
c X = F

An exhaustive analysis of the available structure–activity data for most of the main groups of carcinogens has recently been undertaken by Arcos and Argus and, in part, Wolf (Arcos *et al.*, 1968; Arcos and Argus, 1974). The guiding principle of their work was to establish or emphasize order within the field of chemical carcinogenesis. However, both this and related studies (Clayson, 1962; Ross, 1962) have failed to establish many generally applicable structure–activity principles that are common to the various series of carcinogens. This is probably due more to the vagaries of *in vivo* testing methods than to the absence of any underlying coherence between apparently related series of carcinogens. It is possible that *in vitro* assays may discern these underlying patterns which are negated or at least become obscured *in vivo*, especially when results from different species of test animal or obtained with different test protocols are compared. With this in mind, some broad structure–activity comparisons are outlined below for the main series of carcinogens. Emphasis has been placed upon showing the large range of molecular changes which can be made to some carcinogens without loss of their carcinogenic activity. An attempt has also been made to generate generalized structures for some of the classes of carcinogens described and, when this has not been possible, to indicate the approximate structural boundaries of carcinogenicity within these classes. References to relative potency have generally been avoided. Some of the structural groupings made below may be questioned because of differing modes of action or differing target specificities of chemically related compounds. This does not, however, necessarily nullify such a correlation of chemical similarity. Recently defined carcinogens of which the basic carcinogenicity data are still seriously contested have been omitted from this study, as any conclusions could prove either premature or based on controvertible data. None the less, it is just these areas of concern that require rapid and well planned chemical and *in vitro* evaluation.

*Aromatic amines.*–The various series of carcinogenic aromatic amines, viewed collectively, constitute one of the largest families of carcinogens. For the present purpose this grouping is meant to include the aminobiphenyls, benzidines and aminoazobenzenes, the di- and tri(p-aminophenyl)methanes, the aminostilbenes, some amino derivatives of fluorene, dibenzothiophene and dibenzofuran and finally 2-naphthylamine and its analogues. Despite the reservations outlined above there is a growing body of evidence linking these various series in at least some stages of their respective carcinogenic processes. For example, ring hydroxylation and conjugation is a usual method of detoxification and most have been shown to undergo carcinogenic activation *via* *N*-hydroxylation (Poirier *et al.*, 1967; Miller *et al.*, 1960, 1961, 1966; Anderson *et al.*, 1964; Baldwin and Smith, 1965; Sato *et al.*, 1966; Troll *et al.*, 1965; Boyland and Manson, 1966). Furthermore, in several series the resultant *N*-hydroxy compounds, themselves carcinogens, have been shown to undergo esterification of the *N*-hydroxy group followed by reaction with nuclear material, either purine or protein in origin (Miller and Miller, 1966; Kriek, 1965; Lin *et al.*, *al.*, 1975; Lotlikar *et al.*, 1966). However, the absence of any reliable rules governing activity within these series means that the 4 generic structures (12)–(15) should be considered as representing them when attempting to detect future carcinogens in this class.

(12)

(13)

(14)

(15)

In Structures (12)–(15) the presence of a second amine substituent should be considered as optional, thus, Structure (12) can accommodate both derivatives of 4-aminobiphenyl and benzidine. Similarly, the point of attachment of the first amine substituent is not initially critical; for example, carcinogenicity has been detected in both 1-amino- and 3-aminofluorene (U.S. Public Health Service, 1951; Morris *et al.*, 1960), both of which are positional isomers of the parent carcinogen 2-aminofluorene (14); $X = CH_2$. Structure (13) can accommodate various amino-stilbene and aminoazobenzene carcinogens, and Structure (15) 2-anthramine ($X = Y = CH$) and analogues such as 2-naphthylamine (X and Y absent). Many of the chemicals included in the structures (12)–(15) have been prepared and tested, and many are carcinogenic (Miller and Miller, 1953 *a*, *b*; Miller *et al.*, 1955) when bearing appropriate, but apparently arbitrary, substituents. However, many have not yet been studied, and their non-carcinogenicity cannot be assumed. When considering the nature of the *N*-substituents in Compounds (12)–(15) the complete metabolic equivalence of the following groups should initially be assumed: $NH_2$, NHCOR, NHOH, NOH(COR), N(OCOR′)(COR), NO, $NMe_2$ and $NO_2$. There are a few exceptions to the above. For example, 4-aminoquinoline-1-oxide (16a) is non-carcinogenic, whilst the corresponding 4-nitro- and 4-hydroxylamino compounds (16b and c) are potent carcinogens. However, this is sufficiently rare to be initially ignored. Disguised forms of metabolically activated amine substituents should also be considered as potential carcinogens. For example, Compound (17) a cyclic hydroxamic acid prepared from the carcinogen 4-NQO (16b) (Yaramoi *et al.*, 1975), possesses the N-hydroxy-N-acyl grouping found in the ultimate carcinogenic form of 2-acetylaminofluorene and related carcinogens. Compound (17) must, therefore, be regarded as a potential carcinogen worthy of at least *in vitro* evaluation in a short-term test.

(16) a $X = NH_2$

b $X = NO_2$

c $X = NHOH$

(17)

A further important generalization of Structures (12)–(15) can be made in the light of the established carcinogenicity of various 2-nitrofuryl compounds (Arcos and Argus, 1974), an example of which is the quinazoline (18) (Cohen and Bryan, 1973; Erturk *et al.*, 1971). The 2-nitrothienyl analogue (isostere) (19) of (18) has recently been reported to be carcinogenic whilst the simple thienyl analogue of (19), wherein the $—NO_2$ group is absent, was inactive (Cohen and Bryan, 1973).

Apart from equating the effect of furan and thiophene in such situations, the inactivity of the *des*-nitro compound underlines the importance of the nitro

(18)

(19)

group and, therefore, argues for a connection between both (18) and (19) and the 4-amino/nitro/biphenyl carcinogens. Thiophene and furan may, therefore, be capable of acting as direct replacements for a benzene ring in aromatic-amine carcinogens. Recent metabolic studies on compounds such as (18) and (19) also argue in favour of such a connection (Wang *et al.*, 1975*a*). This connection is made even more viable by the related disclosures that several heterocyclic compounds, such as (20) and (21), are potent carcinogens (Cohen *et al.*, 1975) and mutagens (Wang, *et al.*, 1975*b*; Tazima *et al.*, 1975; Olive and McCalla, 1975).

(20)

(21)

Compound (20) represents a single replacement and compound (21) a double replacement of the benzene rings in benzidine by heteroaromatic rings, each change occurring with a retention of carcinogenicity. It would be logical, therefore, to generalize Structures (12)–(15) further to include *any* heteroaromatic ring system in place of the benzene rings.

To exemplify the implications of the above ideas, consider the hypothetical molecule (22). This compound fits the generalized formula (14) and should, therefore, be initially regarded as a potential carcinogen.

(22)

Despite their apparent generality, Structures (12)–(15) should not be regarded as exclusive. For example, Compounds (24) (Miller *et al.*, 1955) and (25) (Cook *et al.*, 1940) can be regarded as nominal analogues of 2-naphthylamine (Hueper and Wolfe, 1937) (23) which are not covered by Structures (12)–(15) and both are carcinogenic. Further, the terphenyl compound (26) (Miller *et al.*, 1956) a somewhat distant analogue of 4-aminobiphenyl, is not covered by the generic structures, yet is a weak carcinogen.

(23)

(24)

(25)

(26)

*Metabolic considerations based on the arylamine carcinogens.*—Metabolic conversions in this general class of carcinogens have been studied in greater detail than they have in any other class. The carcinogenic activation mechanism has been alluded to above. Ring hydroxylation followed by conjugation is often encountered as a metabolic "detoxification" mechanism in this group of compounds (Arcos and Argus, 1974). However, it does not follow from this that the substitution of a phenolic hydroxyl group in the nucleus of a known carcinogen will render it inactive, because, for example, both 3-hydroxy-4-aminobiphenyl (Gorrod *et al.*, 1968), and 3,3′-dihydroxybenzidine (Baker, 1953), are carcinogenic. Despite these examples, there does exist a general trend towards non-carcinogenicity when a carcinogen of this and other classes is ring-substituted with a polar or lipophobic group, such as hydroxy (OH) or sulphonic acid group (—$SO_3H$). This is illustrated by the sulphonic acids (27) (Spitz *et al.*, 1950) (28) (Rossner, 1937) (29) (Windaus and Rennhak, 1937) and (30) (Kinosita, 1937), which are all non-carcinogenic derivatives of established carcinogens. It is possible that 2 separate mechanisms may be operating to render Compounds (27)–(30) non-carcinogenic. The first, which would probably apply to the benzidine derivative (27), may involve chemical interference with the metabolic carcinogenic activation of the —$NH_2$ function by the sulphonic acid group. Such examples would be expected to be non-mutagenic *in vitro* as well as being non-carcinogenic *in vivo*. The second mechanism, which may apply to Analogues (28) and (29), may depend upon interference with the systemic absorption and distribution of the compound *in vivo*, owing to the physical presence of the polar sulphonic-acid group. Such a mechanism may not automatically interrupt the expected carcinogenic metabolic activation of the basic nucleus should it be allowed to reach the appropriate metabolic site. These compounds may, therefore, be mutagenic *in vitro* yet non-carcinogenic *in vivo*. Whilst non-carcinogenicity is acceptable for a compound, however it may be arrived at, the above considerations may lead to some apparently false predictions of carcinogenic potential by *in vitro* tests for some sulphonic-acid derivatives. It may therefore be equally as unrealistic to accuse an *in vitro* test of producing "false" results in such cases as it would be to assume that all compounds containing a sulphonic-acid group were inherently non-carcinogenic.

(27)

(28)

(29)

(30)

The possibility of *in vivo* metabolism of a compound into smaller fragments, some of which may resemble or actually be carcinogens, is also worth consideration. A well documented example of this is the *in vivo* formation of *o*-aminoazotoluene (32) from Scarlet Red (31) (U.S. Public Health Service, 1951; Stoeber, 1909; Hayward, 1909).

Compound (32) was subsequently tested and shown to be a carcinogen, thus explaining the carcinogenic effect observed for the parent dye (31) (U.S. Public Health Service, 1951; Yoshida, 1932). This example becomes more important with the realization that the liver enzyme(s) "azoreductase", which is held responsible for the carcinogenic activation of Scarlet

(31)

(32)

Red, is also responsible for the metabolic detoxification of other azo carcinogens such as 4-methylaminoazobenzene (33) (Kensler, 1949; Rukzki, 1975). The same enzyme system is, therefore, responsible for the carcinogenic activation of compound (31) and for the carcinogenic deactivation of the related compound (33). This fact alone has serious implications when testing azo compounds in a

(33)

test which incorporates metabolic activation. The microsomal level of the azoreductase enzymes could be critical to both the test response and its likely *in vivo* significance.

Most azo-dyes contain sulphonic-acid groups. However, before all such compounds are regarded as safe (see above) attention should be paid to the eventual position of the sulphonic-acid group after azoreductase-mediated cleavage of the compound. For example, cleavage of the model compound (34) would yield benzidine, and as such may present a potential source of hazard, whilst the model compound (35) would generate the non-carcinogenic 3,3′-disulphonic acid derivative of benzidine.

4-*Nitroquinoline-N-oxide* (4*NQO*) (36*a*).—This compound and its active analogues are generally regarded (Arcos and Argus, 1974) as a distinct group of carcinogens, essentially unrelated to the much larger class of aromatic amines discussed above. Although there are some obvious links with these other carcinogens, such as the increased carcinogenic activity of the derived hydroxyamino compound (36b) (Kawazoe *et al*., 1967; Paul *et al*., 1971), there are good reasons for keeping them as a separate group. Not least amongst these reasons is the surprising inactivity of the corresponding 4-aminoquinoline-N-oxide (36c) (Kawazoe *et al*., 1967) when viewed in the context of other paired carcinogens such as 4-nitro- and 4-amino-biphenyl (WHO/IARC, 1972). Further, susceptibility of the 4-nitro group in 4NQO (36a) to nucleophilic displacement has added a complication to the study of metabolic carcinogen activation in this series not encountered in the others (Arcos and Argus, 1974)

Two main points of relevance to the present study emerge from this series. The first concerns the unusually large number of changes which can be made to

(34) X = H

(35) X = $SO_3H$

(36) a X = $NO_2$
b X = NHOH
c X = $NH_2$

the basic 4NQO structure whilst still retaining carcinogenic activity. Activity is retained in 4-chloroquinoline-N-oxide (Searle, 1965, 1966, 1967, 1968), 4-nitroquinoline (Mori *et al.*, 1969), the 6,7 dimethyl analogue (37) (Lacassagne *et al.*, 1966) and even in quinoline itself (Hirao *et al.*, 1976). Compound (37) represents a ring opened form of 6,7-benzo-4-nitroquinoline-N-oxide (38) and thus the latter compound will probably also prove to be carcinogenic when tested (Arcos and Argus, 1974) (this structural transition is well established amongst the polycyclic aromatic hydrocarbon carcinogens; see later). Similarly, carcinogenicity is retained with "partial" removal of the fused benzene ring of 4NQO (34a). Although the immediately derived 4-nitropyridine-N-oxide is apparently inactive, its 3-methyl analogue (39) is carcinogenic (Araki *et al.*, 1971).

(37)

(38)

(39)

The second area of interest presented by the 4NQO series concerns inhibition of carcinogenicity by steric crowding around the 4-nitro group. Both 9-nitroacridine-N-oxide (40) (Hirao *et al.*, 1976) and 3-methyl-4-nitroquinoline-N-oxide (41) are non-carcinogenic (Kawazoe *et al.*, 1967). In contrast, the methyl-substituted isomer (42), where at least one approach to the nitro group is left unhindered, is carcinogenic (Kawazoe *et al.*, 1967; Nakahara *et al.*, 1958). It is possible that interaction of the 4-nitro group with nitro reductase

(40)

(41)

(42)

enzymes is being sterically inhibited in compounds (40) and (41), and this is clearly related to the proposed steric inhibition of amine–oxidase interaction (Garner *et al.*, 1975) and established non-carcinogenicity (Holland *et al.*, 1974) of 3,3′,5,5′-tetramethylbenzidine referred to earlier. Based on the above findings, Arcos and Argus have predicted (Arcos and Argus, 1974) that 3,5-dimethyl-4-nitropyridine-N-oxide (43) will be non-carcinogenic. Many similar predictions

(43)

could be made in superficially related series, and this steric inhibition of *amino* or *nitro*-mediated carcinogenicity may well prove to be a general, and extremely valuable, inter-series effect.

*Polycyclic aromatic hydrocarbons.*—So many compounds in this series are known carcinogens that any new compound should undergo some form of evaluation before being released for widespread use. Many structure–activity hypotheses have been advanced to explain carcinogenicity, or the lack of it, in these compounds (Arcos *et al.*, 1968; Brookes, 1977; Jerina and Daly, 1977). Two separate lines of thought may make these rather obscure compounds relevant to the large middle ground of chemicals whose structures do not directly resemble any of the established polycyclic carcinogens. The first of these concerns the systematic modification of primary polycylic carcinogens such as 3,4,9,10-dibenzpyrene (44) (U.S. Public Health Service, 1951). It has been found that successive replacements of the fused benzene rings of such compounds with methyl or alkyl groups often retains carcinogenicity. In this way the 2 potent carcinogens 3-methylcholanthrene (45) (U.S. Public Health Service, 1951) and 7,12-dimethylbenz(a)anthracene (46) (U.S. Public Health Service, 1951) and the progressively simpler and weaker carcinogens 8,9,10,11-tetrahydro-7,12-dimethylbenz(a)anthracene (47) (U.S. Public Health Service, 1951) 1,2,3,4-tetramethylphenanthrene (48) (Badger *et al.*, 1942) and 9,10-dimethylanthracene (49), (Lijinsky and Saffiotti, 1965) can be derived.

(44)

(45)

(46)

(47)

(48)

(49)

(50)

(51)

Apart from simulating a benzene ring in the above situations, the methyl groups themselves may play a part in the carcinogenic sequence. For example, the hydroxymethyl compounds (50) and (51) (Yang and Dower, 1975), themselves carcinogens (Boyland, 1969), have been detected as the major metabolites formed when 7,12-dimethyl-1,2-benzanthracene (46) is treated with a liver homogenate from normal rats. None the less, the production of these alcohols is probably an artefact (Brookes, 1977). Whatever their function, methyl groups seem to be instrumental in converting the non-carcinogen anthracene into the carcinogen 9,10-dimethylanthracene (49). Correctly positioned methyl groups, or substituted methyl groups, must, therefore, be considered capable of replacing a fused benzene ring in some carcinogens, and perhaps more important, be capable of transforming other non-carcinogenic polycyclic aromatic compounds into carcinogens (Boyland, 1952). Such an effect can be further disguised by the appropriate and simultaneous isosteric replacement of a fused benzene ring by, for example, a thiophene ring. Many examples of thiophene acting as an equi-active replacement for a fused benzene ring have been observed within this general class of carcinogens (U.S. Public Health Service, 1951; Sax, 1975). Therefore, bringing both of these lines together, 3,4-dimethyldibenzothiophene (52) (Campaigne *et al.*, 1969*a*) should be regarded as a derivative of the weak carcinogen chrysene (53) (U.S. Public Health Service, 1951) and as such should be viewed as potentially carcinogenic and worthy of *in vitro* evaluation.

(52)

(53)

A second area of interest amongst the polycyclic aromatic carcinogens is that concerning their putative relationship to anti-tumour compounds such as ellipticene (54) and olivacine. The structural similarity of ellipticine to the carcinogen 7,12-dimethylbenzanthracene (55) has been noted elsewhere (Campaigne *et al.*, 1969*b*). Furthermore, both of these compounds reduce to even more similar structures by the isosteric replacement of the pyrrolic ring of (54) and the "K-ring" of (55) by thiophene rings giving compounds (56) (Fujiwara *et al.*, 1968) and (57) (Robinson and Tilak, 1947) respectively. Both of these analogues retain their original properties and both have been suggested to act *via* interaction with DNA base pairs (Boyland, 1969; Swan, 1967). The above facts taken in the light of Haddow's paradox (see below) argue for the inclusion of compounds as remote as (54) within the present definition of potential polycyclic carcinogens.

*Anti-tumour compounds and Haddow's paradox.*—Haddow's paradox (Badger *et al.*, 1942; Haddow, 1947) concerns the association between anti-tumour (carcinolytic) and carcinogenic properties in some substances. The aspect of particular relevance to the present study is that a given anti-tumour compound may under special circumstances exhibit a carcinogenic effect. For example, both 4-dimethylaminostilbene (Haddow *et al.*, 1948) and 4-nitroquinoline-N-oxide (Sakai *et al.*, 1955) were originally regarded as anti-tumour compounds, and only later, based on Haddow's paradox, were they tested for carcinogenicity and found to be active. Likewise 4-chloroquinoline-N-oxide possesses both carcinolytic and carcinogenic properties under the appropriate circumstances (Searle, 1965, 1966, 1967, 1968). There are areas where the above concept is self evident, as with the use of alkylating agents in anti-tumour therapy. For example, both cyclophosphamide (Weisburger, 1975) and thioTEPA (U.S. Public Health Service, 1951) have been shown to possess carcinogenic properties, and the nitrosourea anti-tumour agents (Montgomery *et al.*, 1974; Fujiwara *et al.*, 1974) can be associated with the carcinogenicity of N-methyl-N-nitrosourea itself. However, other anti-tumour agents, of which there are many, are less easily associated with known carcinogens. Despite the fact that the basis for labelling these compounds as carcinolytics is often tenuous it is perhaps worthwhile including them in a data bank of potential carcinogens for cross-reference to new compounds being assessed for potential carcinogenicity.

*Nitrosamines, nitrosamides, hydrazines and azoxyalkyl compounds.*—The nitrosamines, along with a variety of nitrosoureas and nitrosoguanidines, form a well established class of carcinogens (Preussmann *et al.*, 1969; Toth, 1975; Magee and Barnes, 1967; Druckrey *et al.*, 1969; IARC, 1972, 1974; Druckrey, 1975). The simplest nitrosamines such as dimethylnitrosamine (58) and nitrosomorpholine (59) have been studied in the most detail; however, relatively exotic nitrosamines such as nitrosofolic acid (60) (Wogan *et al.*, 1975), nitrosoephedrine (Wogan *et al.*, 1975) and the glucitol derivative (61) (U.S.Public Health Service, 1951) are also animal carcinogens. Nitrosamines are formed by the reaction of nitrites with secondary amines over a range of pH conditions (Ziebarth, 1974; Challis and Kyrtopoulos, 1976). These chemical species occasionally occur together in the environment (IARC, 1972. 1974; Tate and Alexander, 1975; Quarles and Tennant, 1975; Rao, 1975) and in some foodstuffs (IARC, 1972, 1974; Shubik, 1975), often under conditions favourable for nitrosamine formation (Ziebarth, 1974; Challis and Kyrtopoulos, 1976). The realization of this fact, and its possible implications, has led to an increase in the study of nitrosamines as carcinogens in laboratory animals and in mutation test systems. Although a clear link between occupational or environmental exposure to nitrosamines and human cancer has not yet been established, it has been demonstrated (Bartsche and Montesano, 1975) that dimethylnitrosamine is activated as a mutagen by both human and rat liver

(58)

(59)

(60)

(61)

microsomes, and consequently it would be prudent to regard nitrosamines as potential human carcinogens at the present time (Montesano, 1975).

The synthesis and properties of the acetylated α-hydroxymethylnitrosamine (62) was recently described (Fahmy *et al.*, 1975*a*, *b*; Wiessler, 1974). This compound has been shown to possess greater mutagenic and carcinogenic properties (Fahmy *et al.*, 1975*b*) than the parent nitrosamine (58), and thus its intermediacy in the metabolic activation of dimethylnitrosamine as a carcinogen seems likely (Druckrey, 1975). It must be pointed out however, that β-hydroxylation also has been suggested as a route to the activation of other analogues such as dibutylnitrosamine (Althoff *et al.*, 1975). If it is assumed that α-hydroxylation is a primary step in the activation of all nitrosamines it should follow that analogues having partially or fully blocked α-positions will be non-carcinogenic, and this is broadly observed. Diphenylnitrosamine is non-carcinogenic in several species (Hashida *et al.*, 1973; Innes *et al.*, 1969), t-butyl ethylnitrosamine (63) is both non-carcinogenic (Druckrey *et al.*, 1963) and non-mutagenic (Pasternak, 1963) and the piperidine derivative (64) is of very low or zero carcinogenicity (Lijinsky and Taylor, 1975) (β-hydroxylation may account for the residual carcinogenicity).

Two further complicating factors must be borne in mind when assessing the carcinogenic risk likely to be associated

(62)

(63)

(64)

with a nitrosamine. The first relates to the ability of tertiary amines such as (65) to undergo *mono*-dealkylation in the presence of sodium nitrite. The derived secondary amine can then react further to produce a nitrosamine (Fiddler *et al.*, 1972). The second hidden problem centres on the possibility that non-carcinogenic nitrosamines such as diphenylnitrosamine may act as transnitrosating agents to other secondary amines, thereby producing a new and potentially carcinogenic nitrosamine. Transnitrosation is a well established phenomenon in aromatic nitrosamines (Buglass *et al.*, 1974; Welzel, 1971) and in some (Johnston *et al.*, 1975) but not all (Buglass *et al.*, 1974) aliphatic nitrosamines. The biological significance of transnitrosation has been discussed elsewhere in the present context (Buglass *et al.*, 1974; Shapley, 1975), and the recently established carcinogenicity of nitrosofolic acid (Wogan *et al.*, 1975) adds weight to this concern

(58)

The nitrosourea (66) and the nitrosoguanidine (67) are representatives of the nitrosamide family of carcinogens (U.S. Public Health Service, 1951; Druckrey, 1975; Ward and Weisburger, 1975), and several derivatives are employed as antitumour agents (Wheeler, 1975; Reed and May, 1975).

Thus, although there is now a much better understanding of structural requirements for the carcinogenicity of

(66)

(67)

nitrosamines, all such compounds should be regarded with caution except in a few well defined cases such as diphenylnitrosamine. Secondary or tertiary amines which are likely to come into contact with nitrites should also be carefully considered. Two further groups of carcinogens are worth consideration in the context of nitrosamine. The first of these is the azoxylalkane group, of which ethyl azoxyethane (Druckrey *et al.*, 1966) (68) the cycasin derivative (69) (Matsumoto *et al.*, 1965; Laqueur, 1964) and perhaps elaiomycin (70) (Ehrlich *et al.*, 1954; Schoental, 1967) are representative. It has been

(68)

(69)

(70)

suggested that the carcinogenicity of both cycasin and dimethylnitrosamine may be expressed *via* a common alkylating intermediate (Miller, 1964; Matsumoto and Higa, 1966), and this has been made more likely by the synthesis of the dimethylnitrosamine intermediate (62), which happens to be isomeric with the cycasin ester (69). Likewise, a similarity between the acute liver toxicity produced by both dimethylnitrosamine and elaiomycin (70) has been noted (Schoental, 1967). The second class of compounds which may be related to the above groups is that of the hydrazines. Most of the derivatives of hydrazine which have been tested, including derivatives as structurally diverse as (71) (Toth and Schimizu, 1974), (72) (Innes *et al.*, 1969), (73) (Toth, 1973) and hydrazine (Biancifiori and Ribacchi, 1962) itself, have been proved experimentally to be carcinogens (Toth, 1975).

In the light of these observations, all derivatives of hydrazine, excluding those

(71)

(72)

(73)

in which the hydrazine group forms part of an aromatic ring system (Colvin, 1969) (*e.g.* cinnolines) should be regarded as potentially carcinogenic (Toth, 1973; Biancifiori and Ribacchi, 1962). Acid hydrazides such as isoniazid (74) have also been shown to be experimental carcinogens (Biancifiori and Ribacchi, 1962; Mori *et al.*, 1960; Miller, 1975;) although the effect in such compounds is probably mediated *via* metabolic transformation to hydrazine (Colvin, 1969).

(74)

(59) (75)

The reason for including hydrazine derivatives in the present group stems from the suggestion of Preussmann *et al.* (1969) that metabolic oxidation of hydrazines to azo or azoxy compounds may precede their carcinogenic response. Further, nitrosomorpholine (59) is reduced *in vitro* by guinea-pig liver microsomes to N-aminomorpholine (75) (Suss, 1965), a derivative of hydrazine. The weak mutagenicity of (75) (Suss, 1965) and the mutagenicity (Lingens, 1964) and carcinogenicity (Toth, 1973) of the closely related 1,1-dimethylhydrazine (73) led to the suggestion that intermediate hydrazine formation was a possible step in the carcinogenic activation of nitrosamines in general (Suss, 1965). However, the oxidative route of activation of hydrazine derivatives is currently the most favoured.

*Alkylating agents*

Several groups of carcinogens elicit their effects *via* the metabolic formation of alkylating species (*e.g.* the nitrosamines and alkylhydrazines). However, a large number of compounds can be grouped under the general heading of alkylating agents. In its simplest and chemical sense this definition is meant to include compounds which are capable of reacting with nucleophiles. However, the aspect of relevance to the present analysis is whether such compounds are capable of acting as biologically *significant* alkylating agents, a distinction not always immediately apparent. Examples of most of the chemical types included within this group can be found among the alkylating agents used in anti-tumour therapy, *e.g.* (76)-(79). These examples happen to be bifunctional, and most are known to react with nuclear DNA (Ross, 1962). In theory, at least, any compound which possesses a chemical leaving group that can irreversibly interact with a nucleophile has carcinogenic potential. However, experience has modified this position to the point that carcinogenicity is usually only associated with discrete and optimally activated members of a particular class (Ross, 1962). For example, the alkylating activity of the carcinogen β-propiolactone (80) (Roe and Salaman, 1955) appears to be strongly dependent upon the ring strain present in this compound, because in the less strained homologue, γ-butyrolactone (81) (Dickens and Jones, 1961) carcinogenicity is absent. Lactones, in fact, form a comparatively small group of carcinogens wherein the structural requirements for carcinogenic activity are partially understood (Dickens, 1964). Activity is most generally associated with unsaturated lactones such as patulin (82) (Dickens, 1964) and aflatoxin $B_1$ (83) (U.S. Public Health Service, 1951) where the carcinogenic action is probably not totally dependent upon the lactone ring. The chemical alkylating agents form too large a group of compounds for any general stance to be adopted when predicting carcinogenicity for a new member, and the situation will not be helped by the possibility that *in vitro* tests might find all such compounds positive. The partition coefficient, chemical half-life and specific nucleophile reactivity of each possible carcinogen of this class will probably determine whether any carcinogenic effects are observed in animals.

(76)

(77)

(78)

(79)

(80)

(81)

(82)

(83)

*Miscellaneous groups*

Several apparently miscellaneous carcinogens can be grouped together because each possesses an activated carbon–carbon double bond which is known to react with biologically occurring nucleophiles. This type of reaction is usually detected by the isolation of cysteine or methionine adducts, the carcinogenic significance of which is uncertain. Such a reaction may only represent a common detoxification pathway, but it could also be taken as an indicator of the established carcinogenicity of these compounds, which is usually mediated *via* a derived epoxide. Obviously not all compounds that react with biologically occurring sulphur compounds are carcinogens (Harington, 1967) but some predictive significance may be attached to the following examples. Arecodine (84) is the suggested carcinogenic principle of betel nuts, and in the rat it is converted to the cysteine adduct (85), amongst other metabolites (Boyland and Nery, 1969; Nery, 1971).

(84)

(85)

(86)

(87)

Similarly, the cysteine adduct (87) has been isolated from the urine of rats dosed with the carcinogen vinyl chloride (86) (Green and Hathway, 1975). Safrole (88) (Innes *et al.*, 1969), and its synthetic metabolite acetoxysafrole (89) (Borchert *et al.*, 1973) are both carcinogenic, and likewise the latter compound has been shown to react with methionine *via* addition to the double bond and replacement of acetate ion, to give the thiomethyl adduct (90) (Borchert *et al.*, 1973). Interestingly, this same derivative (89)

(88)

(89) (90)

was also shown to react with guanosine monophosphate to produce the analogous 0-6 alkylation product, (Borchert *et al.*, 1973) thus paralleling a biologically important reaction with the methionine "marker reaction". A similar arrangement of atoms to that found in (89) (allylic leaving group) is present in the pyrrolizidine alkaloid carcinogens such as heliotrine (91) (Schoental, 1975), although at least 2 alternative mechanisms of carcinogenic action have been proposed for these compounds (Mattocks, 1974; Culvenor *et al.*, 1976). Several classes of anti-tumour agents also fall into this general classification of activated double bonds, *e.g.* the chalcones of type (92) (Dore and Viel, 1974).

(91)

(92)

However, although Michael acceptors appear to offer a potential alkylation site, and therefore could be classed as potential carcinogens, the following is pertinent. The keto-derivative of safrole (93) reacts readily but reversibly with DNA and it was non-carcinogenic when tested alongside compounds (88) and (89) (Wislocki, 1974).

(93)

None the less, acrylonitrile, which is an efficient Michael acceptor has been shown to be carcinogenic (British Industrial Biological Research Association, 1977). In this case it is probable that intermediate formation of a derived epoxide had mediated the observed carcinogenic effect. Potential carcinogenicity was predicted for this compound by an *in vitro* test long before animal carcinogenicity was defined (Venitt *et al.*, 1977).

*General considerations*

The metabolic conversion of a chemical to an active intermediate capable of reacting with DNA will usually be a dynamic and competitive process. Whilst one metabolic sequence may activate a compound, there will usually be several alternative transformations operating at the same time which will deactivate either the test chemical or any derived active intermediates. Therefore, whether or not a *significant* DNA reactivity is obtained for a compound, either *in vitro* or *in vivo*, may depend upon the net balance between activation and deactivation pathways for the compound in a given metabolic situation. The possibility exists, therefore, that structural and metabolic considerations may suggest that a compound will possess DNA reactivity and is therefore a potential carcinogen, yet due to the unforeseen dominance of certain deactivation pathways it may be inactive both *in vitro* and *in vivo*. In other words, many compounds may be classed as potential carcinogens by virtue of the postulated or established existence of an activation pathway in their metabolism, yet only those with a *significant* activation component to their overall metabolism will prove active.

The above considerations will lead to structural analysis over-estimating the number of potential carcinogens within a group of compounds. In contrast, the suppression of metabolic deactivation pathways with appropriate competitive substrates could lead to the accentuation of an otherwise minor activation pathway for a compound, as evidenced by a negative *in vitro* test response, and the production of a positive response. However, to contrive a positive test response for an otherwise inactive compound would be to justify the initial structural concerns at the expense of ignoring the metabolic reality for the compound.

If structural analysis is to be employed as a method of predicting carcinogenicity, the above uncertainties must be clearly recognized. For example, it would be justifiable to be suspicious, initially, of all aromatic nitro compounds because each *may* undergo metabolic transformation to a DNA-reactive hydroxylamine-ester intermediate. However, only those nitro compounds which form a biologically significant level of such an intermediate, probably as evidenced by a positive *in vitro* test response, should be considered as potentially carcinogenic. It therefore follows that a negative *in vitro* response given by structurally suspect compounds will often represent a failure to anticipate correctly the *overall* metabolism of a compound, rather than a failure of structural analysis *per se*. The obvious sensitivity of this approach means that its best place in any screening battery is at the beginning, and that it should be applied even before *in vitro* testing is initiated.

*Concluding comments*

The past few years have witnessed an unprecedented increase both in the number of newly defined carcinogens and in our general awareness of the potential hazard to humans which carcinogens in general may pose. During this period a variety of *in vitro* tests have become generally accepted as being useful contributors to any attempts to detect carcinogens, and a large number of long-term carcinogenicity studies have been either initiated or completed. Moreover, these studies have indicated that carcinogenicity may not be the rare and structurally confined phenomenon it was once thought to be. If this trend continues, or increases, a situation will arise wherein a bewilderingly diverse collection of organic chemicals will be defined as carcinogens for at least one species of animal, a condition which will be exacerbated by a mounting backlog of potential animal carcinogens as defined by *in vitro* assays.

In contrast, if an attempt is made to define areas of maximum potential hazard (*via* structural considerations and human-exposure estimations) and to study these areas in coordinated programmes, a much greater impact on the hypothetical human problem (Higginson, 1969) could be made. It has been suggested that the current lack of a sufficient data-base substantially limits the use of structural analysis in predicting carcinogenicity (Fisher and Fishbein, 1977). This, however, merely emphasizes the points made above. That carcinogenesis and mutagenesis are not completely arbitrary and unpredictable phenomena has been amply demonstrated already; what is lacking is a current willingness to plan future testing in such a way as to generate a sound data-base for future predictions. If this is not undertaken, the situation will develop wherein responsible agencies are forced to react individually to each alert without taking cognizance of structurally related compounds which may also present a potential hazard, or of these newly defined animal carcinogens, which is likely to present a real human hazard. This could lead to a clogging of the very machinery which has been instituted to protect people.

Attempts to devise testing priorities and either discern or generate structure–activity relationships, with an accompanying chemical rationale for the observed *in vivo* or *in vitro* effects which may be associated with a new carcinogen, might enable the current flood of data to be transformed into an ordered attack on the proposed problem.

None the less, it would be misleading to infer that all compounds capable of inducing tumours in animals can be so rationalized and classified. None of the chemical considerations referred to above can explain how, for example, saccharin or phenobarbitone induce tumours in animals, neither do *in vitro* tests find such compounds positive. If such compounds were to be increasingly detected, and if the biological effects they elicit should be considered significant to man, a new and diffuse class of potential carcinogens would have to be recognized, a class not open to the above methods of prediction (Ashby *et al.*, 1978).

REFERENCES

ALTHOFF, J., EAGEN, M. & GRANDJEAN, C. (1975) *J. natn. Cancer Inst.*, **55**, 1209.

ANDERSON, R. A., ENOMOTO, M., MILLER, E. C. & MILLER, J. A. (1964) *Cancer Res.*, **24**, 128.

ARAKI TACHIBANA, M., KOGA, C. & KAWAZOE, Y. (1971) *Gann*, **62**, 325.

ARCOS, J. C. & ARGUS, M. F. (1974) *Chemical Induction of Cancer, IIA.* New York, London: Academic Press.

ARCOS, J. C. & ARGUS, M. F. (1974) *Chemical Induction of Cancer, IIB.* New York, London: Academic Press. pp. 28, 34, 37, 93, 217.

ARCOS, J. C., ARGUS, M. F. & WOLF, G. (1968) *Chemical Induction of Cancer, I.* New York, London: Academic Press.

ASHBY, J., STYLES, J. A., ANDERSON, D. A. and PATON, D. (1978) *Fd. Cosmet. Toxicol.*, **16**, 95.

BADGER, G. M., COOK, J. W., HEWETT, C. L., KENNAWAY, E. L., KENNAWAY, N. M. & MARTIN, R. H. (1942) *Proc. Roy. Soc. B*, **131**, 170.

BADGER, G. M., ELSON, L. A., HADDOW, A., HEWETT, C. L. & ROBINSON, A. M. (1942) *Proc. Roy. Soc. B*, **130**, 255.

BAKER, R. K. (1953) *Cancer Res.*, **13**, 137.

BALDWIN, R. W. & SMITH, W. R. D. (1965) *Br. J. Cancer*, **19**, 433.

BARTSCHE, H. & MONTESANO, R. (1975) *Proc. Am. Soc. Cancer Res.*, **16**, 85.

BERENBLUM, I., Ed. (1974) *Carcinogenesis as a*

*Biological Problem*. Amsterdam: N. Holland Publishing Co.
BERGMANN, F. (1942) *Cancer Res.*, **2**, 660.
BERGMANN, E. D. & PULLMAN, B., Eds. (1969) *Physico-chemical Mechanisms of Carcinogenesis.* Jerusalem: Israel Academy of Sciences and Humanities.
BIANCIFIORI, C. & RIBACCHI, R. (1962) *Nature*, **194**, 488.
BONSER, G. M., BRADSHAW, L., CLAYSON, D. B. & JULL, J. W. (1956) *Br. J. Cancer*, **10**, 539.
BORCHERT, P., WISLOCKI, P. G., MILLER, J. A. & MILLER, E. C. (1973) *Cancer Res.*, **33**, 575.
BOYLAND, E. (1952) *Cancer Res.*, **12**, 77.
BOYLAND, E. (1969) In *Physico-chemical Mechanisms of Carcinogenesis*. E. D. Bergmann and B. Pullman, Eds. Jerusalem: Israel Academy of Sciences and Humanities. p. 33.
BOYLAND, E. & MANSON, D. (1966) *Biochem. J.*, **101**, 84.
BOYLAND, E. & NERY, R. (1969) *Biochem. J.*, **113**, 123.
BRITISH INDUSTRIAL BIOLOGICAL RESEARCH ASSOCIATION (1977*a*) *BIBRA Inf. Bull.*, **16**, 222.
BRITISH INDUSTRIAL BIOLOGICAL RESEARCH ASSOCIATION (1977*b*) *Pesticide, Toxic and Chemical News.*, 25 May, 21.
BROOKES, P. (1977) *Mutation Res.*, **39**, 257.
BUGLASS, A. J., CHALLIS, B. C. & OSBORNE. M. R. (1974) *IARC Scientific Publications*, **9**, 94.
CAMPAIGNE, E., HEWITT, L. & ASHBY, J. (1969*a*) *J. Heterocyclic Chem.*, **6**, 553.
CAMPAIGNE, E., ASHBY, J. & OSBORN, S. W. (1969*b*) *J. Heterocyclic Chem.*, **6**, 885.
CARTER, R. L. & ROE, F. J. C. (1975) *J. Soc. Occup. Med.*, **25**, 86.
CHALLIS, B. C. & KYRTOPOULOS, S. A. (1976) *J. Chem. Soc. Commun.*, **21**, 877.
CLAYSON, B. D. (1962) *Chemical Carcinogenesis.* London: Churchill.
COHEN, S. M. & BRYAN, G. T. (1973) *Fed. Proc.*, **32**, 825.
COHEN, S. M., ERTURK, E., VON ESCH, A. M., CROVETTI, A. J. & BRYAN, G. T. (1975) *J. natn. Cancer Inst.*, **54**, 841.
COLVIN, L. B. (1969) The Metabolism of Various Derivatives of Hydrazine: a Review. *Pharm. Sciences*, **58**, 1433.
COOK, J. W., HEWETT, C. L., KENNAWAY & KENNAWAY, N. M. (1940) *Am. J. Cancer*, **40**, 62.
CULVENOR, C. C. J., EDGAR, J. A., JAGO, M. V., OUTTERIDGE, A., PETERSON, J. E. & SMITH, L. W. (1976) *Chem.-Biol. Interactions*, **12**, 299.
DICKENS, F. (1964) *Br. med. Bull.*, **20**, 96.
DICKENS, F. & JONES, H. E. H. (1961) *Br. J. Cancer*, **15**, 85.
DORE, J. C. & VIEL, C. (1974) *J. Pharm. Belg.*, **29**, 341.
DRUCKREY, H. (1975) *Gann Monogr. Cancer Res.*, **17**, 107.
DRUCKREY, H., PREUSSMANN, R., BLUM, G., IVANKOVIC, S. & AFKHAM, J. (1963) *Naturwissenschaften*, **50**, 100.
DRUCKREY, H., PREUSSMANN, R. & IVANKOVIC, S. (1969) *Proc. N.Y. Acad. Sci.*, **163**, 676.
DRUCKREY, H., PREUSSMANN, R., MATSKIES, F. & IVANKOVIC, S. (1966) *Naturwissenschaften*, **53**, 557.
DUNCAN, K. P. (1975) *Br. J. Cancer*, **32**, 260.
EHRLICH, J., ANDERSON, L. E., COFFEY, G. L., FELDMANN, W. H., FISHER, M. W., HILLEGAS, A. B., KARLSON, A. G., KNUDSEN, M. P., ELSTON, J. K., YOUMANS, A. S. & YOUMANS, G. P. (1954) *Antibiotics Chemother.*, **4**, 338.
ERTURK, E., MORRIS, J. E., COHEN, S. M., VON ESCH, A. M., CROVETTI, A. J., PRICE, J. M. & BRYAN, G. T. (1971) *J. natn. Cancer Inst.*, **47**, 437.
FAHMY, O. G., FAHMY, M. J. &WIESSLER, M. (1975*a*) *Biochem. Pharmac.*, **24**, 1145.
FAHMY, O. G., FAHMY, M. J. & WIESSLER, M. (1975*b*) *Biochem. Pharmac.*, **24**, 2009.
FERGUSON, L. N. (1975) *J. chem. Educ.*, **52**, 688.
FIDDLER, W., PENSABENE, J. W., DOERR, R. C. & WASSERMAN, A. E. (1972) *Nature*, **236**, 307.
FISHER, F. & FISHBEIN, L. (1977) Structure Analysis Utility in Predicting Carcinogenicity Questioned. *Pesticide Tox. Chem. News*, Sept., 15.
FUJIWARA, A. N., ACTON, E. M. & GOODMAN, L. (1968) *J. Heterocyclic Chem.*, **5**, 853.
FUJIWARA, A. N., ACTON, E. M. & HENRY, D. W. (1974) *J. Med. Chem.*, **17**, 392.
GADIAN, T. (1975) *Chem. Ind.*, 821.
GARNER, R. C., WALPOLE, A. L. & ROSE, F. L. (1975) *Cancer Lett.*, **1**, 39.
GORROD, J. W., CARTER, R. L. & ROE, F. J. C. (1968) *Ann. Rep. Br. Emp. Cancer Campaign*, **46**, 5.
GREEN, T. & HATHWAY, D. E. (1975) *Chem.-Biol. Interact.*, **11**, 545.
GRISWOLD, D. P., CASEY, A. E., WEISBURGER, E. K., WEISBURGER, J. H. & SCHABEL, F. M. (1966) *Cancer Res.*, **26**, 619.
HADDOW, A. (1947) *Br. med. Bull.*, **4**, 331.
HADDOW, A., HARRIS, R. J. C., KON, G. A. R. & ROE, E. M. F. (1948) *Phil. Trans. Roy. Soc.*, **241A**, 147.
HARINGTON, J. S. (1967) *Adv. Cancer Res.*, **10**, 248.
HASHIDA, C., URUSHIBARA, S. & AKIYANA, M. (1973) *Tokyo Jikeikai Med. J.*, **88**, 688.
HATHWAY, D., Ed. (1972) *Foreign Compound Metabolism in Mammals, II.* London: The Chemical Society. p. 272.
HATHWAY, D., Ed. (1975) *Foreign Compound Metabolism in Mammals III.* London: The Chemical Society. p. 356.
HAYWARD, E. (1909) *Munch. Med. Wschr.*, **56**, 1836.
HIGGINSON, J. (1969) *Present Trends in Cancer Epidemiology.* In *Canadian Cancer Conference.* Ed. J. F. Morgan. Oxford: Pergamon Press. p. 40.
HIRAO, K., SHINOHARA, Y., TSUDA, H., FUKUSHIMA, S., TAKAHASHI, M. & ITO, N. (1976) Carcinogenic activity of quinoline on rat liver. *Cancer Res.*, **36**, 329.
HOLLAND, V. R., SAUNDERS, B. C., ROSE, F. V. & WALPOLE, A. L. (1974) *Tetrahedron*, **30**, 3299.
HOWE, J. R. (1975) *Lab. Practice*, 457.
HUEPER, W. C. (1955) *Archs Indust. Hlth*, **11**, 494.
HUEPER, W. C. & WOLFE, H. D. (1937) *Am. J. Path.*, **13**, 656.
IARC (1972) *IARC Scientific Publications* 3. Lyon: IARC.
IARC (1974) *IARC Scientific Publications* 9. Lyon: IARC.
INNES, J. R. M., ULLAND, B. M., VALERIO, M. C., PETRUCELLI, L., FISHBEIN, L., HART, E. R., POLLOTTA, A. J., BATES, R. R., FALK, H. L., CART, J. J., KLEIN, M., MITCHELL, I. & PETERS, J. (1969) *J. natn. Cancer Inst.*, **42**, 1101.
JERINA, D. M. & DALY, J. W. (1977) In *Drug*

*Metabolism*. D. V. Parke and L. R. Smith, Eds. London: Taylor and Francis. p. 13.
JOHNSTON, T. P., McCALEB, G. S. & MONTGOMERY, J. A. (1975) *J. Med. Chem.*, **18**, 104.
KAWAZOE, Y., TACHIBANA, M., AOKI, K. & NAKAHARA, W. (1967) *Biochem. Pharmacol.*, **16**, 631.
KENSLER, C. J. (1949) *J. Biol. Chem.*, **179**, 1079.
KINOSITA, R. (1937) *Jap. Path. Soc. Trans.*, **27**, 665.
KRIEK, E. (1965) *Biochem. biophys. Res. Commun.*, **20**, 793.
LACASSAGNE, A., BUU-HOI, N. P., ZAJDELA, F., HOEFFINGER, J. P. & JACQUIGNON, P. (1966) *Life Sci.*, **5**, 1945.
LAQUEUR, G. L. (1964) *Fed. Proc.*, **23**, 1337.
LEWIN, R. (1976) *New Scientist*, **69**, 168.
LIN, J. K., MILLER, J. A. & MILLER, E. C. (1975) *Cancer Res.*, **35**, 844.
LINGENS, F. (1964) *Z. Naturforsch.*, **20b**, 151.
LIJINSKY, W. & SAFFIOTTI, U. (1965) *Ann. Ital. Derm. Clin. Sper.*, **19**, 34.
LIJINSKY, W. & TAYLOR, H. W. (1975) *Int. J. Cancer*, **16**, 318.
LOTLIKAR, P. D., SCRIBNER, J. D., MILLER, J. A. & MILLER, E. C. (1966) *Life Sci.*, **5**, 1263.
MAGEE, P. N. & BARNES, J. M. (1967) *Adv. Cancer Res.*, **10**, 164.
MATSUMOTO, H. & HIGA, H. H. (1966) *Biochem. J.*, **98**, 20c.
MATSUMOTO, H., NAGAHAMA, T. & LARSON, H. O. (1965) *Biochem. J.*, **95**, 13c.
MATTOCKS, A. R. (1974) *Excerpta Medica International Congress Series*, **350**, .
MAYER, V. W. & FLAMM, W. G. (1975) *Mutation Res.*, **29**, 295.
McCANN, J., CHOI, E., YAMASAKI, E. & AMES, B. N. (1975) *Proc. natn. Acad. Sci., U.S.A.*, **72**, 5135.
MILLER, C. T. (1975) *Br. J. Prevent. Soc. Med.*, **29**, 64.
MILLER, E. C., FLETCHER, T. L., MARGRETH, A. & MILLER, J. A. (1962) *Cancer Res.*, **22**, 1002.
MILLER, E. C., LOTLIKAR, P. D., PITOT, H. C., FLETCHER, T. & MILLER, J. A. (1966) *Cancer Res.*, **26**, 2239.
MILLER, E. C. & MILLER, J. A. (1966) *Pharmacol. Rev.*, **18**, 805.
MILLER, E. C., SANDIN, R. B., MILLER, J. A. & RUSCH, H. P. (1956) *Cancer Res.*, **16**, 525.
MILLER, E. C., SANDIN, R. B., MILLER, J. A. & RUSCH, H. P. (1956) *Cancer Res.*, **16**, 525.
MILLER, J. A. (1964) *Fed. Proc.*, **23**, 1361.
MILLER, J. A., CRAMER, J. W. & MILLER, E. C. (1960) *Cancer Res.*, **20**, 950.
MILLER, J. A. & MILLER, E. C. (1948) *J. expl Med., Med.*, **87**, 139.
MILLER, J. A. & MILLER, E. C. (1953*a*) *Advanc. Cancer Res.*, **1**, 353.
MILLER, J. A. & MILLER, E. C. (1953*b*) *Advanc. Cancer Res.*, **1**, 356.
MILLER, J. A. & MILLER, E. C. (1971) Editorial. *J. natn. Cancer Inst.*, **47**, 5.
MILLER, J. A. & MILLER, E. C. (1975) *Mutation Res.*, **33**, 25.
MILLER, J. A., SANDIN, R. B., MILLER, E. C. & RUSCH, H. P. (1955) *Cancer Res.*, **15**, 88.
MILLER, J. A., WYATT, C. S., MILLER, E. C. & HARTMANN, H. (1961) *Cancer Res.*, **21**, 1465.
MONTESANO, R. (1975) Are Nitrosamines Human Carcinogens? In *Environmental Carcinogens*. Meeting of the Royal College of Pathologists, London, 12 Nov. 1975.
MONTGOMERY, J. A., MAYO, J. G. & HANSCH, C. (1974) *J. Med. Chem.*, **17**, 477.
MORI, K., KONDO, M., TAMURA, M., ICHIMURA, H. & OHTA, A. (1969) *Gann*, **60**, 663.
MORI, K., YASUNO, A. & MATSUMOTO, K. (1960) *Gann*, **51**, 83.
MORRIS, H. P., VELAT, C. A., WAGNER, B. P., DAHLGARD, M. & RAY, F. E. (1960) *J. natn. Cancer Inst.*, **24**, 149.
MUNN, A. (1974) *J. Soc. Occup. Med.*, **24**, 90.
NAKAHARA, W., FUKUOKA, F. & SAKAI, S. (1958) *Gann*, **49**, 33.
NATIONAL INSTITUTE OF OCCUPATIONAL SAFETY AND HEALTH (1975) NIOSH List of Suspected Carcinogens. *Fed. Reg.*, **40**, 26390.
NERY, R. (1971) *Biochem. J.*, **122**, 503.
OLIVE, P. L. & McCALLA, D. R. (1975) *Cancer Res.*, **35**, 781.
PASTERNAK, L. (1963) *Acta. bid. med. ger.*, **10**, 436.
PAUL, J. S., MONTGOMERY, P. & LOUIS, J. B. (1971) *Cancer Res.*, **31**, 413.
POIRIER, L. A., MILLER, J. A., MILLER, E. C. & SATO, K. (1967) *Cancer Res.*, **27**, 1600.
PREUSSMANN, R., DRUCKREY, H., IVANKOVIC, S. & HODENBERG, A. V. (1969) *Ann. N.Y. Acad. Sci.*, **163**, 697.
PREUSSMAN, R., DRUCKREY, H., IVANKOVIC, S. & HODENBURG, A. V. (1969) *Ann. N.Y. Acad. Sci.*, **163**, 701.
PURCHASE, I. F. H., LONGSTAFF, E., ASHBY, J., STYLES, J. A., ANDERSON, D., LEFEVRE, P. A. & WESTWOOD, F. R. (1976) *Nature*, **264**, 624.
QUARLES, J. M. & TENNANT, R. W. (1975) *Cancer Res.*, **35**, 2637.
RAO, G. S. (1975) Am. Chem. Soc. 170th Abstr. of Papers Med. Sect. 60.
REED, D. J. & MAY, H. E. (1975) *Life Sci.*, **16**, 1263.
ROBINSON, R. & TILAK, B. D. (1947) *24th Ann. Rep. Br. Emp. Cancer Campaign*, 126.
ROE, F. J. C. & SALAMAN, M. H. (1955) *Br. J. Cancer*, **9**, 177.
ROSE, F. L. (1974) *Proc. roy. Soc. Med.* (*B*), **185**, 159.
ROSS. W. C. J. (1962) *Biological Alkylating Agents*. London: Butterworth.
ROSSNER, N. (1937) *Ztschr. f. physiol. Chem.*, **249**, 267.
RUKZKI, E. (1975) *Contact Dermatitis*, **1**, 247.
SAKAI, S., MINODA, K., SAITO, G., KAGI, S. A., UENO, A. & FUKUSKA, F. (1955) *Gann*. **46**, 605.
SANDIN, R. B., MELBY, R., HAY, A. S., JONES, E. C. & MILLER, J. A. (1952) *J. Amer. Chem. Soc.*, **74**, 5073.
SATO, K., POIRIER, L. A., MILLER, J. A. & MILLER, E. C. (1966) *Cancer Res.*, **26**, 1678.
SAX, N. I. (1975) *Dangerous Properties of Industrial Materials*, 4th ed. Van Nostrand Reinhold.
SCHOENTAL, R. (1967) *Advanc. Cancer Res.*, **10**, 236.
SCHOENTAL, R. (1975*a*) *Cancer Res.*, **35**, 2020.
SCRIBNER, J. D. (1975*b*) Editorial. *J. natn. Cancer Inst.*, **55**, 1035.
SEARLE, C. E. (1965) *Ann. Rep. Br. Emp. Cancer Campaign*, **43**, 391.
SEARLE, C. E. (1966) *Ann. Rep. Br. Emp. Cancer Campaign*, **44**, 231.
SEARLE, C. E. (1967) *Ann. Rep. Br. Emp. Cancer Campaign*, **45**, 271.
SEARLE, C. E. (1968) *Ann. Rep. Br. Emp. Cancer Campaign*, **46**, 246.
SEARLE, C. E. (1970) *Chem. Br.*, **6**, 5.
SHAPLEY, D. (1975) *Science*, **191**, 268.
SHUBIK, P. (1975) *Cancer Res.*, **35**, 3475.

SPITZ, S., MAGUIGAN, W. H. & DOBRINER, K. (1950) *Cancer*, **3**, 789.
STOEBER, H. (1909) *Munch. Med. Wschr.*, **56**, 129.
SUSS, R. (1965) *Z. Naturforsch.*, **19b**, 714.
SWAN, J. M. (1967) *Aust. J. Science*, **29**, 435.
TATE, R. L. & ALEXANDER, M. (1975) *J. natn. Cancer Inst.*, **53**, 327.
TAZIMA, Y., KADA, T. & MURAKAMI, A. (1975) *Mutation Res.*, **32**, 55.
TOTH, B. (1973) *J. natn. Cancer Inst.*, **50**, 181.
TOTH, B. (1975) *Cancer Res.*, **35**, 3693.
TOTH, B. & SCHIMIZU, H. (1974) *J. natn. Cancer Inst.*, **52**, 241.
TROLL, W., RINDE, E. & TESSLAR, A. (1965) *Proc. Amer. Assoc. Cancer Res.*, **6**, 65.
U.S. PUBLIC HEALTH SERVICE (1951) Survey of Compounds which have been Tested for Carcinogenic Activity. *U.S. Public Health Publ.*, **149** and Suppls. Washington, D.C.: U.S. Govt. Printing Office.
VENITT, S., BUSHELL, C. T. & OSBORNE, M. (1977) *Mutation Res.*, **46**, 241.
VESTERBERG, O. (1975) *Int. Arch. Occup. Environm. Hlth*, **35**, 89.
WALPOLE, A. L. & WILLIAMS, M. H. C. (1958) *Br. med. Bull.*, **14**, 141.
WANG, C. Y., CHIU, C. W. & BRYAN, G. T. (1975*a*) *Biochem. Pharmacol.*, **24**, 1563.
WANG, C. Y., MURAOKA, K. & BRYAN, G. T. (1975*b*) *Cancer Res.*, **35**, 3611.
WARD, J. M. & WEISBURGER, E. K. (1975) *Cancer Res.*, **35**, 1938.
WEISBURGER, E. K. (1975) *J. clin. Pharmac.*, p. 5.
WELZEL, P. (1971) *Chem. Ber.*, **104**, 808.
WHEELER, G. P. (1975) Mechanism of Action of Nitrosoureas. *Handbook of Experimental Pharmacology, Vol.* **38** (2), p. 65.
WHO/IARC (1972) *Evaluation of Carcinogenic Risk of Chemicals to Man.* WHO/IARC Monogr. Lyon: WHO/IARC. p. 74.
WHO/IARC. Lyon: WHO/IARC.
WIESSLER, M. (1974) *Angew. Chem.*, **86**, 817.
WILSON, R. H., DEEDS, F. & COX, A. J. (1941) *Cancer Res.*, **1**, 595.
WINDAUS, A. & RENNHAK, S. (1937) *Ztschr. f. physiol. Chem.*, **249**, 256.
WISLOCKI, P. (1974) *On the Proximate and Ultimate Carcinogenic Metabolites of Precarcinogens.* Ph.D. thesis, University of Wisconsin.
WOGAN, G. N., PAGLIALUNGA, S., ARCHER, M. C. & TANNENBAUM, S. R. (1975) *Cancer Res.*, **35**, 1981.
YANG, S. K. & DOWER, W. V. (1975) *Proc. natn. Acad. Sci., USA*, **72**, 2601.
YARAMOI, T., NODA, H. & NARRARA, H. (1975) *Tetrahedron*, **31**, 945.
YOSHIDA, T. (1932*a*) *Tokyo Igaku Zasshi*, **46**, 2398.
YOSHIDA, T. (1932*b*) *Trans. Japan. Path. Soc.*, **22**, 193, 934.
ZIEBARTH, D. (1974) *IARC Scientific Publications* 9. Lyon: IARC, 137.